# A Noise Trimming and Positional Significance of Transposon Insertion System to Identify Essential Genes in *Yersinia pestis*

**DOI:** 10.1038/srep41923

**Published:** 2017-02-06

**Authors:** Zheng Rong Yang, Helen L. Bullifent, Karen Moore, Konrad Paszkiewicz, Richard J. Saint, Stephanie J. Southern, Olivia L. Champion, Nicola J. Senior, Mitali Sarkar-Tyson, Petra C. F. Oyston, Timothy P. Atkins, Richard W. Titball

**Affiliations:** 1 Biosciences, University of Exeter, Exeter, EX4 4QD, UK; 2 DSTL, Porton Down, Salisbury, SP4 0JQ, UK; 3 Marshall Centre for Infectious Disease Research and Training, School of Pathology and Laboratory Medicine, University of Western Australia, Perth, Australia

## Abstract

Massively parallel sequencing technology coupled with saturation mutagenesis has provided new and global insights into gene functions and roles. At a simplistic level, the frequency of mutations within genes can indicate the degree of essentiality. However, this approach neglects to take account of the positional significance of mutations - the function of a gene is less likely to be disrupted by a mutation close to the distal ends. Therefore, a systematic bioinformatics approach to improve the reliability of essential gene identification is desirable. We report here a parametric model which introduces a novel mutation feature together with a noise trimming approach to predict the biological significance of Tn5 mutations. We show improved performance of essential gene prediction in the bacterium *Yersinia pestis*, the causative agent of plague. This method would have broad applicability to other organisms and to the identification of genes which are essential for competitiveness or survival under a broad range of stresses.

Genome wide mutagenesis, coupled with next generation sequencing (massively parallel sequencing) technology, has opened a range of opportunities to identify new associations between bacterial phenotypes and genotypes. This approach typically uses a transposon, such as Tn5, which can become randomly integrated into target sites across the genome. By sequencing the DNA flanking the transposon, the insertion sites can be identified. High throughput sequencing allows thousands or even millions of events to be simultaneously mapped^1^,^2^. Depending on the type of transposon used and the methodology employed to sequence the flanking DNA, the combined mutagenesis and sequencing technology has been termed Tn-seq (transposon sequencing), INSeq (insertion sequencing), HITS (high-throughput insertion tracking by deep sequencing) or TraDIS (transposon-directed insertion site sequencing). These transposon sequencing methodologies have been reviewed^1^.

Whilst these different approaches have a broad range of applications, the most common relies on negative selection of a population under stress. Consequently it has been possible to identify genes that are necessary for growth *in vitro* or *in vivo*^1^,^2^,^3^,^4^,^5^,^6^,^7^,^8^. These genes are typically called essential genes because their inactivation results in a fitness disadvantage within an otherwise wildtype population. Essential genes, or more likely their products, are attractive targets for the development of novel therapeutics^9^,^10^,^11^,^12^.

A challenge when dealing with datasets from global mutagenesis studies is interpreting the output^13^,^14^. In theory, under saturation mutagenesis conditions, all target sites in the genome should be mutated. Under negative selection, genes with a fitness disadvantage, evidenced as reduced transposon insertions within the gene of interest, are likely to be essential^1^. The measurement of reduced fitness before and after exposure to a stress has been used to compare populations of mutants, for example to identify genes required for survival and growth *in vivo* compared to genes required for survival and growth *in vitro*^15^,^16^,^17^. However, this method is more difficult to apply to the identification of essential genes, since there is no comparator group. To resolve this problem some workers have assumed that transposon insertion occurs at a similar frequency at all of the possible insertion sites in the genome, and therefore by identifying genes with a reduced frequency of transposon insertions, it is possible to identify essential genes^16^,^18^,^19^,^20^,^21^. The algorithm termed TraDIS^2^,^3^ further develops the idea that essential genes can be identified on the basis of transposon insertion frequencies. TraDIS models transposon distribution using a combination of two Gamma distributions^2^ where a zero-mode (low mean) Gamma distribution is composed of essential genes, and insertion sites of non-essential genes follows a non-zero-mode (large-mean) Gamma distribution. More recently a Hidden Markov model (HMM) has been used to predict gene essentiality^18^,^22^. It was developed to examine mutant libraries which are produced by Mariner transposition. The essentiality of a TA (DNA sequence) site (and therefore potentially the accommodating gene) depends on two factors, the transposon insertion count of the TA site and the essentiality status of the preceding TA site. Further work of a HMM has led to software called TRANSIT, where the densities were estimated using a Bayesian approach^23^.

However, it may also be important to take account of the positional significance of mutations, i.e. transposon insertion at different positions may have varying power to disrupt gene function. Mutations in functionally non-essential regions of otherwise essential genes may be tolerated if they do not compromise the function of the gene product^14^. The possibility that transposon insertions at the 3′ end^6^,^14^ or both 5′ and 3′ ends^8^,^24^ of genes do not affect the biological activity of the gene product has been recognised in a numbers of studies. In the case of 5′ insertions, expression of the mutated genes may be driven by a promoter sequence in the transposon. Because there is no suitable model to consider the positional significance of a mutation, arbitrary filtering has sometimes been used. For instance, insertions beyond the interval between 5% and 85% of the open reading frame^25^, 25%^26^ or within 20% of either end of the gene^27^ have been considered unlikely to disrupt gene function. The software package ESSENTIALS^14^ uses a non-parametric density estimation approach^28^ for essential gene prediction. After insertions at the 3′ end of genes had been discounted, LOESS regression^29^ was used to correct insertion counts, i.e. using regression fitted values to replace the original insertion counts genome-wise. The corrected insertion counts of each individual gene were summed into a single numeric value – the measured insertion count per gene^30^. The log_2_ fold changes between expected insertion counts and measured insertion counts were calculated using edgeR^31^ and were treated as a mutation feature. ESSENTIALS used the first local minimum near zero in an estimated density as the threshold for separating essential genes from non-essential genes. These arbitrary settings work well in these specific studies, but may have limited utility in other experimental systems. Therefore the question of how to deal with the distal effects of mutations remains challenging.

We considered an integrative impact of insertions on gene essentiality, i.e. extracting a mutation feature based on insertions per gene. Each transposon insertion is weighted and integrated to the mutation feature. The weighting process takes into account the insertion sites in relation to the distal ends per gene. Each insertion has a significance value contributing to gene mutation depending on two factors, i.e. how close it is to both distal ends of a gene and how often the insertion site has been hit by a transposon. We have developed a parametric model to integrate all insertions per gene and thus used it for gene essentiality prediction.

A second problem associated with sequencing data analysis is noise. Several studies have reported the presence of low-frequency sequencing events, or noise, which do not faithfully indicate the transposon insertion site. These are variously attributed to sequencing errors or mis-assignment of reads^8^,^24^. In some studies the elimination of this noise has been based on the arbitrary setting of a noise threshold^8^,^24^. We have addressed the noise problem in our study by removing insertions of a very low frequency in individual genes using the tight cluster algorithm to quantitatively remove low insertion counts.

Integrating noise trimming and essential gene identification, we have devised a new approach to the analysis of transposon Tn5 mutagenesis data. We have successfully applied this approach to identify essential genes in *Yersinia pestis*, the aetiological agent of plague.

The major benefit of this work is to bring a new concept for identifying essential genes, which was ignored in previous work. The concept is the recognition of positional significance of transposon insertion sites regarding to the distal ends. It means how far an insertion site is from the distal ends (5′ or 3′). This means that insertions in a gene are weighted in terms of their contribution to the capability of disrupting the function of the gene, depending where these insertions are in the gene. Using this method, we can overcome the difficulty of arbitrarily setting a threshold for discarding transposon insertions within proximal and distal regions before identifying essential genes.

## Results and Discussion

### Three types of essential genes

Before discussing the results, we briefly introduce three types of essential genes. A Type I essential gene is a gene with no transposon insertion. A Type II essential gene is a gene with insertion count lower than a threshold which is determined by a tight clustering algorithm. A Type III essential gene is a gene which has insertion count larger than the threshold, but its transposon insertions were mainly found at proximal and distal ends.

### Nine million sequencing reads from replicate cultures map to about one million transposon insertion sites

We first created a library of over 1 million mutants in *Y. pestis* strain CO92 using an EZ-Tn5 transposome. We used Southern blotting of DNA isolated from 34 randomly picked colonies to confirm that the transposon was inserted randomly into the genome. This library was cultured on three separate occasions (input1, input2 and input3) as biological replicates. DNA was isolated and high-throughput sequencing was carried out to map the transposon insertion sites. We obtained 20.8, 14.6 and 21.8 million total sequence reads respectively from these samples – Supplementary Table S1. The sequence reads were filtered for the presence of the transposon sequence providing 16.4, 11.9 and 17.3 million reads.

When mapped to the *Y. pestis* genome we identified 3.5, 1.7 and 3.8 million transposon insertions. Therefore, we had a dataset of almost 9 million (9,047,401 totalled from 3 samples) which represents 15.8% of the total sequencing reads and 19.8% of the reads containing transposon sequence (Supplementary Table S1). The loss of sequencing reads was mainly due to sequencing errors. The reads were mapped to 330,017, 251,995, and 330,050 insertion sites respectively in the 3 replicates (total 912,062) and the number of insertion sites within genes was between 191,425 and 253,102 in the 3 samples (Table 1). The experimentally determined median distance respectively between insertion sites was 7, 9, and 7 base pairs in the 3 data sets (Supplementary Fig. S1) corresponding well with the theoretical mean distance between insertion sites of 14, 18 and 14 base pairs.

We found that less than 10% of the transposon insertion sites were adjacent to TA sequences in the genome, indicating that Tn5 is less likely to target the TA dinucleotides than Mariner transposons and hence shows that HMM pipelines^18^ may not be suitable for analysing our data. For instance, in input1, there were 252,548 insertions sites in ORFs, of which only 23,077 (9.1% of 252,548 sites) were found beside the TA sequences in the genome. The maximum percentage that inserted beside the TA sequences in the genome was 9.6% in input2. This indicated that TA-based essential gene prediction tools, such as used in Palace’s work^18^, will not properly identify essential genes for our experiment because over 90% of insertions may be discarded. Meanwhile the total number of TA sequences in the genome was also not significantly rich. The number of TA sites was even fewer than the number of insertion sites, for instance, there were only 195,145 TA sites but 330,017 insertions in input1. This is because Tn5 will not target TA sites. Instead this transposon will target GC rich regions as studied previously^32^.

### Type I essential genes

In spite of the density of coverage, some genes lacked any transposon insertions and we termed these genes as Type I essential genes. A Type I essential gene was thus a gene in which no transposon insertion was found. There were 56, 155 and 122 Type I essential genes in three replicate samples respectively.

### Distal end transposon insertions

We examined the patterns of transposon insertions at the distal ends of genes. We did not find any transposon insertions exactly at the distal ends. We thus extended the search of transposon insertions at the distal regions (5% from the distal ends) in two ways, i.e. genome-wise and gene-wise. For a genome-wise search, we examined whether the transposon insertion distribution at the distal ends was random. For a gene-wise search, we examined how often genes had a transposon inserted at their distal regions. The results are shown in Supplementary Table S2. The genome-wise search shows that the transposon insertion distribution at the distal end regions was generally random. The proportion of transposon insertion was 5.43%, 5.5% and 5.39% at the 3′ region for the three samples and was 5.73%, 6.02% and 5.76% at the 5′ region. All the percentages were not very different from 5%. However, the proportion of genes that had transposon insertions at the distal end regions was low. For instance, only 4, 14, and 11 of 3885 genes were found to have transposon insertions at the 3′ region in the three samples respectively. This equates to only 0.1%, 0.4% and 0.3%, much less than 5%. At the 5′ region, only 23, 58, and 50 of 3885 genes were found to have insertions in the three samples respectively. The percentages were still low, being 0.6%, 1.4% and 1.3%, again less than 5%. In addition, only 36, 86 and 74 of 3885 genes were found to have transposon insertions at either distal end regions in the three samples respectively. Based on these figures, the numbers of genes hit by the transposon at both distal regions are 9, 42 and 13. This gene-wise search shows that more genes could be potentially considered as essential genes in addition to Type I essential genes.

### Transposon insertion counts distribution is bimodal

**Figure 1:**
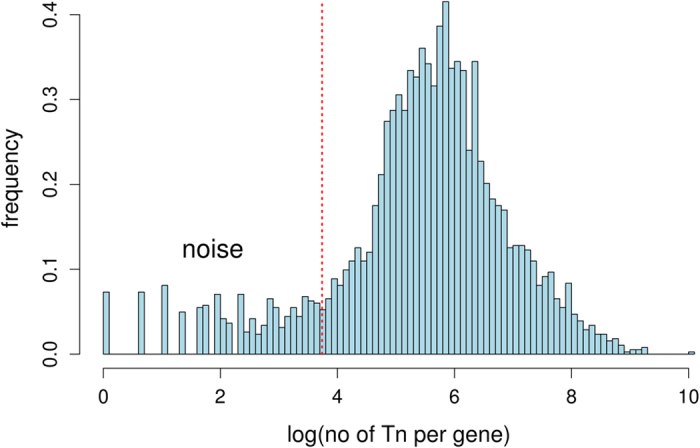
Noise trimming of the input1 dataset. The horizontal axis represents the log of the number of transposon insertions per gene. The vertical axis stands for the frequency of the log of transposon insertion number per gene. The vertical dotted line indicates the threshold corresponding to a critical *p* value 0.05. Genes whose insertion counts were below this threshold were treated as Type II essential genes.

Figure 1 shows the distribution of the logarithm (count) for input1, where count indicates the number of transposon insertions per gene (see Supplementary Fig. S2 for input2 and input3). Because insertions follow a bimodal distribution, we trimmed noise insertions using a tight cluster approach^33^. It separated genes into two clusters. The cluster of genes with greater insertion counts was a major (tight) cluster. The cluster of genes with low transposon insertion counts were treated as Type II essential genes. If a *p* value returned by the tight cluster algorithm was less than 0.05 (critical *p* value), the corresponding gene was treated as a Type II essential gene.

### Relative distance for positional significance

The identification of a Type II essential gene does not consider where insertions are in a gene. We referred to a Type III essential gene as a gene which had a transposon insertion number greater than the threshold set to identify Type II essential genes, but these insertions were mainly located at distal regions and therefore may not disrupt gene function. Supplementary Fig. S3 shows two examples where the upper plot of insertion pattern implies a potential essential gene and the lower plot of insertion pattern implies a gene which is unlikely to be essential.

We proposed a new measurement called Relative Distance (RD) for considering the positional contribution to gene mutation. It was calculated based on the relative distance of a transposon insertion site to the distal ends of a gene. Therefore, one RD was for one insertion. If a transposon insertion was close to either distal end of a gene, its RD was approaching zero. If a transposon insertion was close to the middle base pair of a gene, its RD was approaching one. To determine whether RDs can be used to discriminate between essential and non-essential genes, we used simple statistics (mean) to demonstrate the utility of RDs for separating essential genes from non-essential genes. The RDs were averaged for each gene. The density (histograms) for all three samples (Supplementary Figs S4–S6) show that the RDs of essential and non-essential genes had different distributions. The mean RDs of essential genes showed a left-skewed density towards zero. The density of the mean RDs of non-essential genes, showed a bell-shaped distribution centred at approximately 0.5.

### Mutation features follow a bimodal distribution

**Figure 2:**
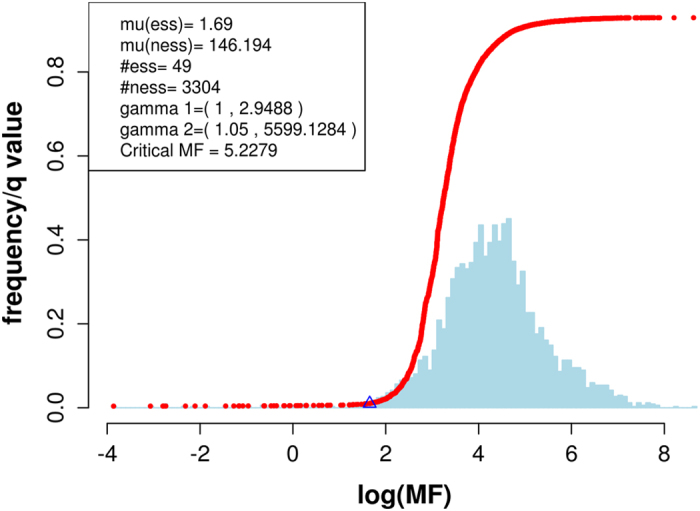
Prediction of essential genes for input1 using DEM. The curve shows the relationship between log MF values and the corresponding false discovery rates (*q* values). The triangle indicates the boundary of separation between essential genes and non-essential genes. Bars in blue represent the density of the log MF values. The horizontal axis stands for log value of MF and the vertical axis stands for the frequency and *q* values.

As mentioned above, individual genes would likely have multiple insertions, hence multiple RDs. Therefore we cannot use a single RD value as a predictor. Integrating RD values per gene into a variable is therefore a feature extraction process^28^,^34^,^35^. The mutation Feature (MF) was calculated based on the convolution of RD values gene-wise on our noise-trimmed data in our work. How to calculate the MF values is explained in Methods and Materials. Plotting MF values as a histogram revealed a broadly bimodal distribution. Therefore genes with small MF values are potentially Type III essential genes – Fig. 2 and Supplementary Fig. S7. Using the Gamma mixture approach, i.e. a mixture of two Gamma densities allowed us to identify additional essential genes which we have termed Type III essential genes.

### The correlation between mutation feature and insertion counts as well as insertion sites

We compared our MF values with the count feature used in ESSENTIALS and the site feature used in TraDIS. Supplementary Fig. S8 shows the correlation patterns for these features in the three samples respectively. All features (MF, insertion counts and insertion sites) were scaled using the natural logarithm. The correlation coefficient was between 0.91 and 0.97.

### Using mutation feature for predicting Type III essential genes

The algorithm proposed in this paper for predicting Type III essential genes is based on MF values calculated for noise-trimmed data. We have termed this algorithm the Distal Effect Model (DEM). Figure 2 shows a prediction of *Y. pestis* essential genes based on MF values. The mean log MF of the predicted Type III essential genes was 1.69 and the mean log MF of the predicted non-essential genes was 146.194. The MF separating the essential from non-essential genes was 5.23, based on a *q* value (false discovery rate) of 0.01. The curve (we refer to it as a decision curve) represents the relationship between the log MF values and the *q* values. The critical (log) MF value was the point (the triangle in Fig. 2) at which essential genes were separated from non-essential genes. Supplementary Fig. 7 visualises the relationship between log MF and the *q* value for the input2 and input 3 samples, and all display monotonic relationships between *q* values and log MF values.

### Consistency of *Y. pestis* essential genes between samples

**Figure 3:**
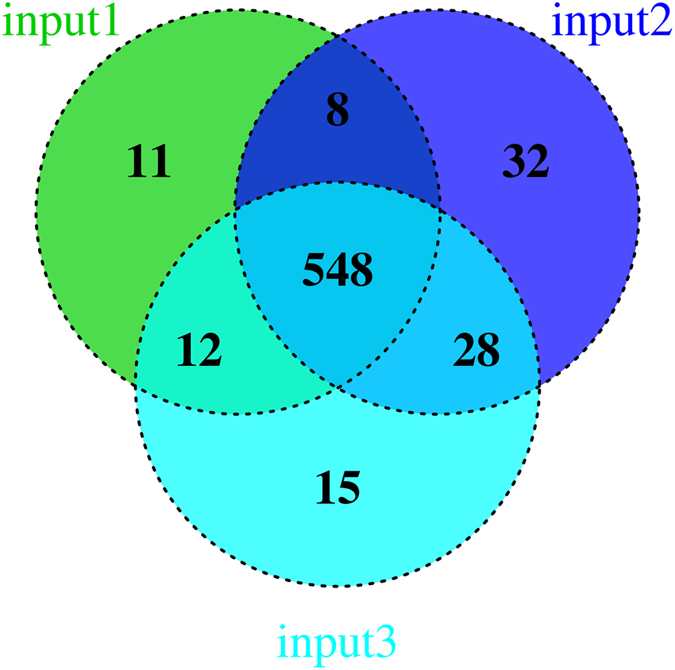
A Venn diagram of all essential genes predicted by our system for the three samples. Essential genes predicted by our system for the three samples. They include all three types of essential genes.

Supplementary Table S3 shows the prediction result using our system (including the identification of all types of essential genes) for the *Y. pestis* input1, input2 and input3 samples. The number of Type I essential genes was 56, 155 and 122 respectively. After noise trimming we identified 415–474 Type II essential genes. Finally, based on the MF feature we identified 46–52 Type III essential genes using the DEM. In total, we identified 579, 616, and 603 essential genes in three samples and 548 genes were common to all three samples (Fig. 3 and Prediction List at http://ecsb.ex.ac.uk). We applied the chi-square test to determine the co-incidence between samples. Type II, Type III and total essential genes show good consistency between samples. The *p* values of the chi-square test were 0.12, 0.83 and 0.56 – Supplementary Table S3. Therefore the distributions of Type II, Type III and total essential genes across the three samples did not show a significant variation.

### Essential gene distribution in the *Y. pestis* chromosome

**Figure 4:**
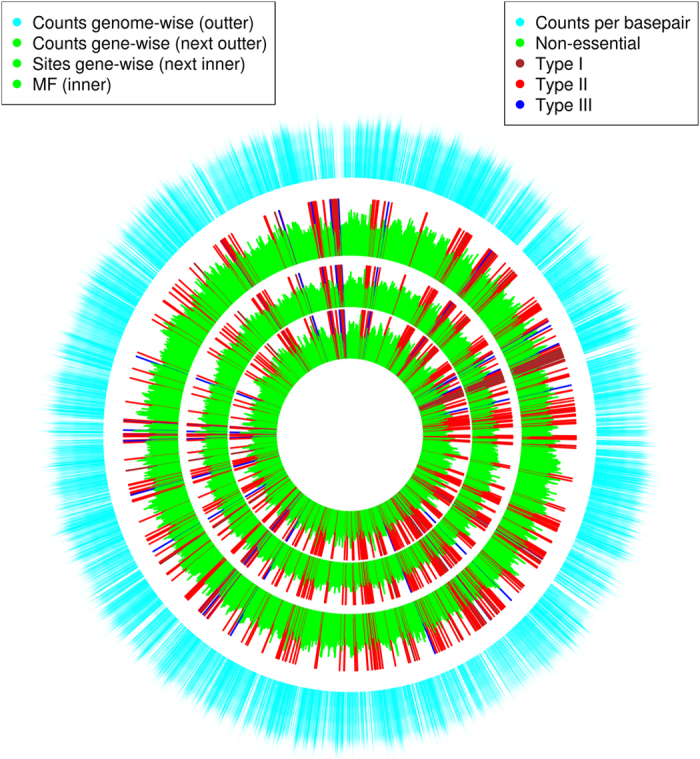
Locations of 548 essential genes identified in the *Y. pestis* chromosome. Moving out from the centre the layers show; MF values; transposon insertion sites per gene for all genes; insertion counts per gene for all genes; transposon insertion counts per base pair genome-wise. Brown bars indicate Type I essential genes, red bars represent Type II essential genes and blue bars represent Type III essential genes.

Figure 4 shows a map of essential genes in the genome. The four layers are composed of four distributions, i.e. MF distribution (inner layer), insertion site distribution (next inner layer), gene-wise insertion count distribution (next outer layer) and genome-wise insertion count (outer layer). The three inner layers are gene-wise, therefore we included the positions of three types of essential genes. We conducted the Kolmogorov–Smirnov test to examine whether the predicted essential genes are randomly distributed in the genome. The Kolmogorov–Smirnov test was conducted between a uniformly distributed position vector across the genome and a vector of the start positions of essential genes. The distribution of the start base-pairs of genes did not follow a normal distribution. The *p* values of the Shapiro-Wilk test of normality were all less than 2.2e-16 by the R function *shapiro.test* for both raw and logarithm start base-pairs. The test showed that only Type III essential genes were randomly distributed throughout the genome - Supplementary Table S4. All *p* values of the test for Type I and Type II essential genes were less than 0.05. Therefore, Type I and Type II essential genes were not randomly distributed throughout the genome. Supplementary Fig. S9 shows the functional analysis of Type I essential genes. It can be seen that most Type I essential genes belong to the category of translation. Supplementary Fig. S10 shows the functional analysis of Type II essential genes. In addition to the translation category, which is still the richest, the oxidation-reduction process is also rich in genes. This may explain the non-randomness of these two types of essential genes, but the mechanism needs further biological examination.

### Comparison of our approach with other essential gene predictors

We first compared the predictions using our system (including all three types of essential gene prediction) with the predictions of TraDIS and ESSENTIALS using two methods. The first method measured the coincidence between the lists of essential genes predicted in the three samples - Table 2. Our system provided 85.1% coincidence between samples. Using ESSENTIALS on the noise-trimmed data the coincidence rate was 75.8% and using TraDIS on the noise-trimmed data it was 79.1%. However, using ESSENTIALS on the non-noise-trimmed data the coincidence rate was 14.5% and using TraDIS on the non-noise-trimmed data the coincidence rate was 75.7%.

The low coincidence rate using ESSENTIALS on non-noise-trimmed data reflected the way in which it uses the first local minima of a density function of corrected insertions as the threshold. If all data are included, the local minima of the density function of corrected insertion counts would move to a low threshold. This is because there might be several clusters within the insertion counts data. Without any human intervention, the algorithm will automatically select a first local minima close to the original, i.e. 0. Therefore, the algorithm will identify a far smaller proportion of the essential genes. Due to randomness of the insertion counts across samples, such local minima in a density function will be uncertain. Therefore, different samples have very different first local minima values, hence very different predictions of essential genes. This explains why the coincidence rate was so low using non-noise-trimmed data for ESSENTIALS. TraDIS on the other hand builds up a mixture of two Gammas to reveal two clusters of data (for examples see Fig. 2 and Supplementary Fig. S7) even if the data includes noise. However, the separation of clusters was less pronounced using the noise-trimmed data - Supplementary Fig. S11. This explains why, compared to our algorithm, TraDIS over-predicted essential genes using noise-trimmed data and indicates that TraDIS would provide a better analysis of data without noise trimming the input data. Supplementary Fig. S12 shows a Venn diagram of essential genes predicted for non-noise-trimmed data by TraDIS (A) and ESSENTIALS (C), and for noise-trimmed data by TraDIS (B) and ESSENTIALS (D). It can be seen that both predicted more essential genes when a noise removal process was used. The prediction of TraDIS on the noise-trimmed data was unlikely to be true because it predicted nearly one third of the total number of genes to be essential. The prediction of ESSENTIALS on the data without noise removal was also unlikely to be true because it predicted too few essential genes. In addition, compared with ESSENTIALS, TraDIS delivered better predictions in terms of the coincident rate, i.e. three sets of predictions of TraDIS were more similar.

The second method comparison method we used was to compare the predicted essential genes in *Y. pestis* with the Database of Essential Genes (DEG)^36^, which includes essential gene data from a variety of bacterial species - Table 2. We found that 82% of the predicted essential genes in *Y. pestis* using our system had previously been reported as essential in other bacterial species. In comparison, 32.5% (without noise-trimming) or 51.7% (with noise-trimming) of genes predicted as essential using TraDIS or ESSENTIALS were found in DEG.

Because our data does not fit the condition of TA-based essential gene prediction using HMM approach, we compared our predictions with the HMM predictions, i.e. in Palace’s work^18^. Supplementary Fig. S13 shows the comparison. The comparison was carried out only for genes with gene symbols because the two experiments displayed different probe set names. It can be seen that the two predictions have about 41% coincidence. The discrepancy is mainly due to the fact that the two studies were carried out under different conditions. We then continued to analyse the genes which were identified as essential genes by our algorithm but not identified as essential genes by HMM in Palace’s work^18^. As shown in Supplementary Fig. S13, 17 Type I essential genes, 137 Type II essential genes and 12 Type III essential genes predicted by our algorithms were not seen in the HMM prediction list produced by the Palace’s work. Surprisingly, all these 17 Type I essential genes have no transposon insertion at all. They are certainly essential genes in our experiment, but missed by the HMM model. Supplementary Fig. S14 shows the transposon insertion patterns for 5 Type III essential genes identified by our algorithm but missed by the HMM predictions. It can be seen that they all have a few transposon insertion sites towards the distal ends. In addition, all of them were also seen in the DEG library^36^. We have compared all three types of essential genes which were missed by the HMM model against the DEG library and we found that 29% of these Type I essential genes, 39% of these Type II essential genes, 42% of these Type III essential genes were found in the DEG library.

### Genes with proximal and distal end insertions

We now examined the genes which only have insertions at proximal and distal ends. What we needed to examine was how these genes were treated in prediction using different algorithms. Supplementary Table S5 shows how these genes were analysed by our algorithm. It can be seen that the 3′ region had lesser number of essential genes identified compared with the 5′ region. This is not surprising because of the analysis in the previous section “Distal end transposon insertions”, where we showed that 3′ insertions were less frequent than 5′ insertions. All genes with 5% distal regions of transposon insertions were identified as Type II or Type III essential genes. In addition, most were identified as Type II essential genes. This is because the insertion count at the distal regions was low in these genes. Supplementary Table S6 shows how these genes were analysed by ESSENTIALS and TraDIS. It can be seen that both ESSENTIALS and TraDIS were not able to make perfect predictions of these genes although it is not completely sure they are essential genes. ESSENTIALS performed even worse than TraDIS. For instance, our algorithm identified all 36 of these genes in input1. However, TraDIS identified 31 of them and ESSENTIALS identified 5 of them only. Supplementary Fig. S15 shows the transposon insertion pattern for the gene YPO3718 (pgi) which was not predicted as an essential gene by TraDIS and ESSENTIALS, but identified as an essential gene by our algorithm. It means that no Type III essential genes were predicted by ESSENTIALS. It can be seen that all insertions were located at the 3′ region. The insertion count is not significantly high.

### The relationship between three types of essential genes and TraDIS and ESSENTIALS

Supplementary Fig. S16 shows the relationship between the three types of essential genes predicted by our algorithm and the predictions of ESSENTIALS and TraDIS. It can be seen that all Type I essential genes were predicted by ESSENTIALS and TraDIS because they don’t have any transposon insertions. The Type II essential genes had a better overlap with TraDIS than ESSENTIALS. For instance, in input1, 333 Type II essential genes were predicted as essential genes by TraDIS, but only 79 Type II essential genes were predicted by ESSENTIALS. In terms of Type III essential genes, TraDIS showed certain overlap, i.e. 7, 11, and 3, but ESSENTIALS showed no overlap with Type III essential genes. In other words, all Type III essential genes cannot be predicted by ESSENTIALS.

### Validation of Type III gene essentiality

We next randomly selected eight Type III genes predicted to be essential by our system and tested them experimentally. A rhamnose-inducible, glucose-repressible copy of the gene was provided for *in trans* complementation. The target gene was then inactivated in the chromosome by λ-red recombinase mutagenesis. Addition of rhamnose resulted in expression of the plasmid-borne copy of the gene, which could then be repressed by removal of rhamnose and addition of glucose. Growth could be followed as optical density of liquid cultures, or by the formation of colonies on solid media. Of the eight genes predicted by the algorithm, all were shown to be essential (Table 3) and included YPO3439 which encodes a hypothetical protein. This protein has not previously identified as essential in any microorganism. The *trmD, ispG* and *spoT* genes were essential for growth on agar but not for growth in broth - Supplementary Fig. S17. It is possible that very low levels of gene expression, even in the presence of glucose repression, allow limited growth which can be detected in broth but not on agar.

## Conclusion

In this paper, we have devised a system for gene mutation analysis from massively sequenced transposon data. The system we have devised has been used to analyse data generated using Tn5 transposon mutagenesis. It is possible that our approach would also be applicable to data generated using other transposons, but this requires further investigation. Genes are classified into two categories – mutational and non-mutational based on a series of identification steps. First, Type I essential genes are identified when establishing the SAM file. Second, the use of the tight cluster approach identifies Type II essential genes. Third, a novel mutation feature has been introduced to classify Type III essential genes. The basic idea for identifying Type III essential genes, which is different to the current algorithms, is to model the impact of transposon insertion sites with respect to the two distal ends of genes on essentiality. This is because a transposon insertion site near either distal end of a gene may not disrupt gene function as shown in the literature. To address this we have introduced a novel idea, relative distance (RD). It is a normalised distance between a transposon insertion site in a gene and two distal ends of the gene. The smaller the RD is, the less likely the transposon is to disrupt the function of an essential gene. Because each gene may have more than one transposon insertion site, hence more than one RD, all RDs in a gene are required to be integrated. If all RDs are small, the function of a gene may not be disrupted. If some RDs are small but some are large, the function of the gene may still be disrupted. We have used a simple convolution method for the integration based on an exponential density function estimated genome wise to derive a mutation feature. Based on the derived mutation feature, our algorithm used a parametric model to penalise the impact of the distal ends transposon insertions on gene essentiality. Through the validation of our predictions against DEG, we show that this newly devised algorithm outperformed existing algorithms, which do not model distal effect well, for the prediction of essential genes.

## Methods and Materials

### Generation of mutants

Bacterial strains used in this study are listed in Supplementary Table S7. *Y. pestis* strains were cultured in blood agar base (BAB) broth or BAB agar supplemented with hemin (0.025%) at 28 °C. Strains of *Escherichia coli (E. coli*) were cultured in Luria-Bertani (LB) broth. When required, media was supplemented with kanamycin (25 μg ml^−1^), trimethoprim (100 μg ml^−1^) and chloramphenicol (25 μg ml^−1^). L-rhamnose (0.02%) and L-glucose (0.1%) were added as appropriate for the validation of targets. A library consisting of over 1 million mutants was constructed in *Y. pestis* CO92 by random mutagenesis using the EZ-Tn5 < kan-2 > Tnp transposome kit (Epicentre) according to the manufacturer’s instructions. *Y. pestis* was cultured in BAB broth at 28 °C and was made electrocompetent by sequential washes in 10% glycerol. Parameters defined previously for the electroporation of *Y. pestis* to high efficiency were applied^37^. After electroporation, cells were allowed to recover for 2 hours in BAB broth prior to plating on BAB-hemin agar for the enumeration of cell numbers and selective BAB-hemin plates containing kanamycin. Mutants were washed from the plates and pooled into batches of 200,000, before combining to create the final transposon library.

### Sequencing of mutants

The transposon library was cultured on three separate occasions in BAB broth at 28 °C overnight, then genomic DNA was extracted using the Gentra Puregene kit (Qiagen). The gDNA was fragmented to <500 base pairs (bp) using 2 × 15 minute cycles at 4 °C in a BioRuptor sonicator (medium intensity, 30 s on/90 s off). The NEBNext DNA library preparation kit for Illumina (NEB) was used, as per the manufacturer’s instructions, to end repair, A-tail and ligate adapters to the fragments. The adapters used were Ind_Ad-T and Ind_Ad-B (The upper part of Supplementary Table S8), which were annealed prior to use. Parallel PCR reactions were set up with 10 μl JumpStart 10× buffer, 6 μl MgCl_2_, 2 μl 10 mM dNTPS, 0.6 μl 100 μM PE_PCR_V3.3 primer, 0.6 μl 100 μM Yp_EzTn_PCR primer, 1 μl JumpStart Taq DNA polymerase and 28.8 μl nuclease-free water per reaction. Primer sequences are listed in the lower part of Supplementary Table S8. The reactions were amplified at 94 °C for 2 minutes, [94 °C for 30 seconds, 60 °C for 20 seconds, 72 °C for 30 seconds] for 22 cycles, 72 °C for 10 minutes, then held at 12 °C. PCR products were pooled and ethanol precipitated before being size selected on a 2% agarose/1 × TBE gel. Agarose blocks corresponding to 350–500 bp were excised, and the DNA extracted using a Qiagen MinElute Gel Extraction kit as per the manufacturer’s instructions. The DNA was quantified by qPCR and on an Agilent BioAnalyzer before being submitted for sequencing as 100 bp single end reads on an Illumina HiSeq 2500 standard model.

Raw sequencing reads were screened for the transposon sequence TTGAGATGTGTATAAGAGACAG. The screened transposon sequencing reads were then mapped to the reference genome (AL590842) using BWA^38^. Rather than calculating the number of transposon insertions (or number of transposon insertion sites) per gene as a variable, we will use our proposed method in this paper to derive a new variable for essentiality prediction.

Supplementary Table S1 details the data process in the stages of transposon sequence recognition, mapping, and insertion sites recognition. In total, there were 57.2 million raw sequencing reads. 45.8 million (80.1%) were transposon sequences. Nine million (19.7%) transposon sequences were mapped to the genome (AL59084). When using BWA^38^, the error rate was set at 2. We increased the error rate to 5. However, there is little change on mapping rate. This may be due to the PCR error. For three samples, 56, 155, and 122 out of 3883 genes had no insertion. They were treated as Type I essential genes. Among these Type I essential genes, 48 were shared by three samples. That input2 and input3 have more Type I essential genes may be because these two samples have less saturated sequencing depth.

### Validation of mutants

Targets selected for validation of the essentials list were placed under the control of a rhamnose-inducible promoter system using a method described previously^39^. Briefly, the open reading frame was cloned into the low copy number plasmid vector pBADrha, under the regulatory control of the *rhaB* gene promoter and accessory genes rhaS/R, before transformation into *Y. pestis* containing the λ-red recombinase helper plasmid, pAJD434. The chromosomal copy of the gene was then replaced by the open reading frame encoding kanamycin resistance using λ-red mutagenesis^40^ before curing of pAJD434 by growth in BAB supplemented with kanamycin (25 μg/ml), rhamnose (0.02%) and CaCl2 (2.5 mM) for 40 hours at 37 °C. Final constructs were tested for mutation, the presence of virulence plasmid and absence of pAJD434 by PCR using the oligonucleotides listed in Supplementary Table S9. *Y. pestis* mutant strains were validated by growth in BAB broth supplemented with rhamnose (0.02%) to induce, or glucose (0.1%) to repress, expression from P_rhaB_. Mutant strains of *Y. pestis* were grown overnight at 28 °C in BAB broth supplemented with kanamycin. This growth in the absence of rhamnose was necessary to titrate reserves of the target protein prior to testing, which for some mutants required a second passage. Bacteria were pelleted to facilitate concentration before addition to BAB broth supplemented with rhamnose or glucose as above to a starting A_590_ of 0.06. Growth curves were performed (x6) in a 96-well format using a Multiskan FC incubated plate reader as described previously^39^. Alternatively, essentiality was confirmed on solid media. *Y. pestis* mutant strains grown overnight at 28 °C with shaking, in either BAB broth or BAB supplemented with 0.1% glucose, were adjusted to an A_590_ of 0.1 prior to performing 10-fold serial dilutions in BAB. Aliquots of 10 μl were pipetted onto BAB agar plates supplemented with 0.02% rhamnose or 0.1% glucose before incubation at 28 °C.

### Systematic approach for detecting essential genes

The process of classifying genes as essential or non-essential genes is to map a mutation feature of a gene to a phenotype (the mutation status of the gene, i.e. essential or not). Suppose MF values of all genes can be modelled in a density function, a mapping function can be constructed using a statistical learning approach such as a maximum likelihood training procedure used in the previous works, i.e. TraDIS^2^,^3^. The constructed mapping function is used to classify a gene as mutational (non-essential) or non-mutational (essential).

Before estimating a mapping function, the challenge is how to define a mutation feature to quantify the genotype cause of phenotype. Such a process is referred to as feature extraction in machine learning^28^,^34^,^35^ and it aims to find the best feature that can describe an experiment in terms of genetic property of essential genes. The success of estimating a good mapping function highly relies on how well a mutation feature is extracted.

We thus defined a novel mutation feature (essentiality score) which can well represent the mutation information inherent in genes. As we know, little work so far has paid attention to this. Mutation prediction is complex because of variable insertion sites, variable insertion counts and variable gene length. An insertion site in a gene determines how likely it is to disrupt the function of the gene. The closer an insertion site is to the distal ends, the less likely it is to disrupt the gene function as discussed in the literature. The insertion count determines the strength of gene function disruption. The greater the insertion count, the more likely it is that the insertion disrupts gene function. This concept has been well utilised in the previous algorithms^2^,^14^. Variable gene length casts a complexity that the impact of insertion varies among genes, in which we examined how often genes possessed transposons in their distal end regions. In other words, a mutation feature must reflect the heterogeneous contribution of these three factors to gene mutation. The significance of mutation of a gene should depend on where an insertion site is and how insertion distributes within a gene. Without the negative selection under a stress, the null hypothesis is that a transposon may be inserted into a genome randomly and uniformly across genes as well as base pairs. However, due to the negative selection, different genes - even different base pairs - will have variable insertion sites and counts. Positional significance of transposon insertion is therefore important and is one of the novelties this work proposes. Accurately integrating them into a mutation feature is thus the key for implementing an effective mapping function between a mutation feature and a phenotype variable.

This system therefore includes everything from sequencing data to the final product of the analysis, which is a list of essential genes. The whole process (workflow) is composed of 6 steps. *Step 1*: sequencing reads with a designed transposon were kept and the transposon was chopped from these reads. These kept reads were referred to as transposon sequences. *Step 2*: the transposon sequences were mapped to a reference genome. This step produced SAM files. *Step 3*: the SAM file was used to calculate transposon insertions per gene. This step had two major outputs. First, RD values for all genes were calculated. Second, Type I essential genes were identified. *Step 4*: noise trimming using the tight cluster approach^33^. This step identified Type II essential genes. *Step 5*: mutation features were calculated gene-wise and DEM was used to identify Type III essential genes. At this step, a list of essential genes can be delivered. *Step 6*: DEG verification and visualisation.

### Relative distance to the distal ends – implementation of positional significance of insertion

Having understood that distal transposon insertion may not disrupt gene function, we combined the site dimension information with gene length information to generate a meaningful dimension in which the distal end effect can be considered at the first instance. We introduced a novel idea called relative distance to the distal ends (RD) – Eq. 3 in Supplementary: The distal effect model. It was defined as one minus the absolute distance between an insertion site and the middle base pair residue of a gene divided by half gene length. By doing so, we can reveal the significance of mutational position (insertion site) quantitatively. RD is between zero and one. If an insertion site is approaching the distal ends, RD is approaching zero. Otherwise, it is approaching one. Each gene then has a RD vector for being transformed to a mutation feature value.

### Mutation feature

We implemented a convolution model to integrate RD values for each individual gene. We estimated an exponential density for all RD values genome-wise. This density function will further penalise the distal effect. The mutation feature was defined as a convolution between RD values and the estimated exponential density function gene-wise, see Eq. S5 in Supplementary: The distal effect model.

### Distal Effect Model algorithm

After a mutation feature was derived, we employed a mixture of two Gammas for gene mutation classification based on mutation feature values gene-wise, following TraDIS^2^,^3^. A maximum likelihood training procedure was used to estimate model parameters. TraDIS used the likelihood value to identify essential genes. We used a statistically more sound method for decision-making, i.e. converting the posterior probability derived from the mixture models to a false discovery rate for decision-making^41^. In addition, TraDIS only used insertion sites for the prediction, but we considered positional significance of transposon insertions in genes. Genes were classified as non-mutational ones if their false discovery rates were less than 0.01, a critical *q* value meaning 1% false discovery rate control. Supplementary: The distal effect model details the RD definition, MF definition and DEM algorithm.

### Benchmark algorithms

We used ESSENTIALS and TraDIS for comparison. We did not use the fitness approach because there is no *a priori* knowledge which gene should be essential and the fitness approach performed poorly in a previous study^18^. We did not use HMM model because our data can have transposons inserted everywhere rather than just at TA sites.

### Data

Three raw sequencing data (FASTQ) are at http://ecsb.ex.ac.uk.

### Code

The R scripts and executable C code are in the web site: http://ecsb.ex.ac.uk.

### Prediction List

The predicted essential genes for three samples: http://ecsb.ex.ac.uk.

## Additional Information

**How to cite this article**: Yang, Z. R. *et al*. A Noise Trimming and Positional Significance of Transposon Insertion System to Identify Essential Genes in *Yersinia pestis. Sci. Rep.* **7**, 41923; doi: 10.1038/srep41923 (2017).

**Publisher's note:** Springer Nature remains neutral with regard to jurisdictional claims in published maps and institutional affiliations.

## Supplementary Material

Supplementary Information

## Figures and Tables

**Table 1:** Mutant analysis.

	input1	input2	input3
site.ALL	330,017	251,995	330,050
site.ORF	252,548	191,425	253,102
tn.follow.TA.ALL	32,368	25,826	32,479
tn.follow.TA.ORF	23,077	18,341	23,248
TA.ALL	195,145	195,145	195,145
TA.ORF	69,656	69,656	69,656

“ALL” stands for the statistics across the whole genome. “ORF” stands for the statistics within ORFs, i.e. genes. “site” stands for the number of transposon insertion sites (mutants). “TA” stands for the base pair sequence (TA) in the genome.

**Table 2:** Comparison of our DEM essential gene prediction algorithm with the performance of other prediction algorithms.

	Noise trim	Total predicted essential genes	Coincidence rate between samples	Coincidence rate with DEG
Our system	Yes	548	85.1%	82.0%
TraDIS	Yes	1034	79.1%	32.4%
ESSENTIALS	Yes	499	75.8%	51.7%
TraDIS	No	342	75.7%	32.5%
ESSENTIALS	No	62	14.5%	51.2%

**Table 3:** Genes validated as essential in this study.

Gene predicted as essential	Phenotype in this study
*murA*	Essential in broth assay
*accA*	Essential in broth assay
*yidC*	Essential in broth assay
*fbaA*	Essential in broth assay
*YPO3439*	Essential in broth assay
*trmD*	Essential on solid media, not in broth
*ispG*	Essential on solid media, not in broth
*spoT*	Essential on solid media, not in broth
