# Supplementary material for: A Noise Trimming and Positional Significance of Transposon Insertion System to Identify Essential Genes in Yersinia pestis
Source: Sci Rep. 2017 Feb 6;7:41923. doi: 10.1038/srep41923 (PMC5292949; doi:10.1038/srep41923)
Supplement: Supplementary Information [file srep41923-s1.doc]

**A Noise Trimming and Positional Significance of Transposon Insertion System to Identify Essential Genes in *Yersinia* *pestis***

Zheng Rong Yang^1^, Helen L Bullifent^2^, Karen Moore^1^, Konrad Paszkiewicz^1^, Richard J Saint^2^, Stephanie J Southern^2^, Olivia L Champion^1^, Nicola J Senior^1^, Mitali Sarkar-Tyson^2^, Petra CF Oyston^2^, Timothy P. Atkins^2^, Richard W Titball^1^

^1^ Biosciences, University of Exeter, Exeter, EX4 4QD, UK.

^2^ DSTL, Porton Down, Salisbury, SP4 0JQ UK.

**Supplementary**


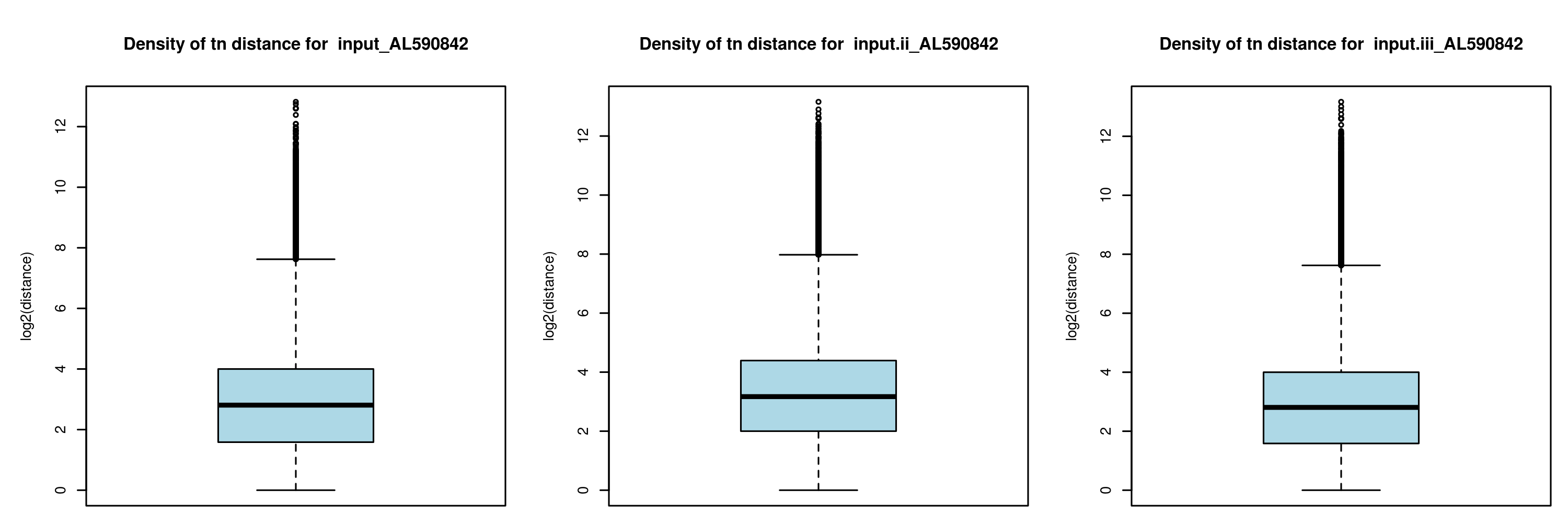


**Fig S1**. The box plots of log2 transposon distances for the three samples. Left panel: input1; Middle panel: input2; Right panel: input3.


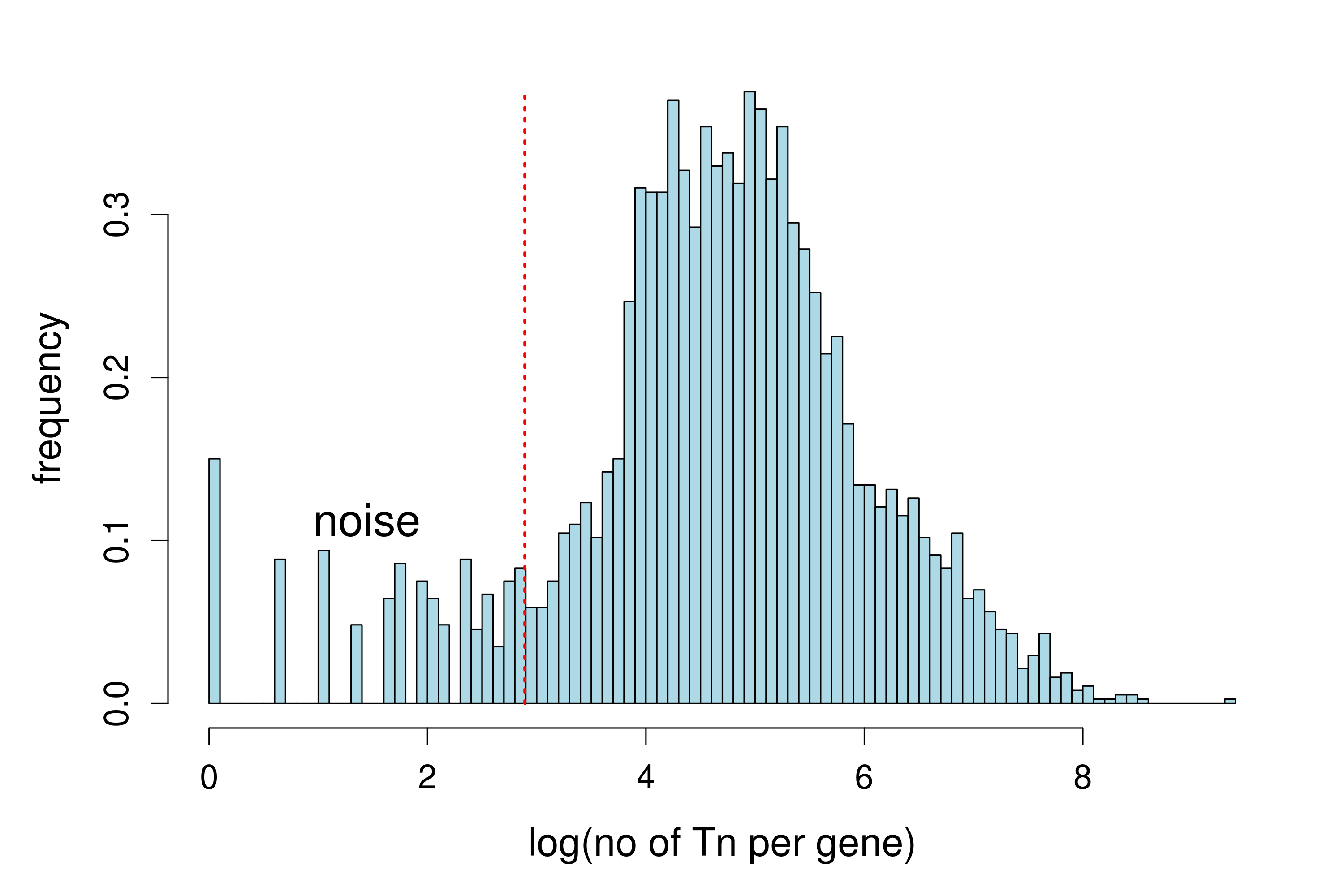

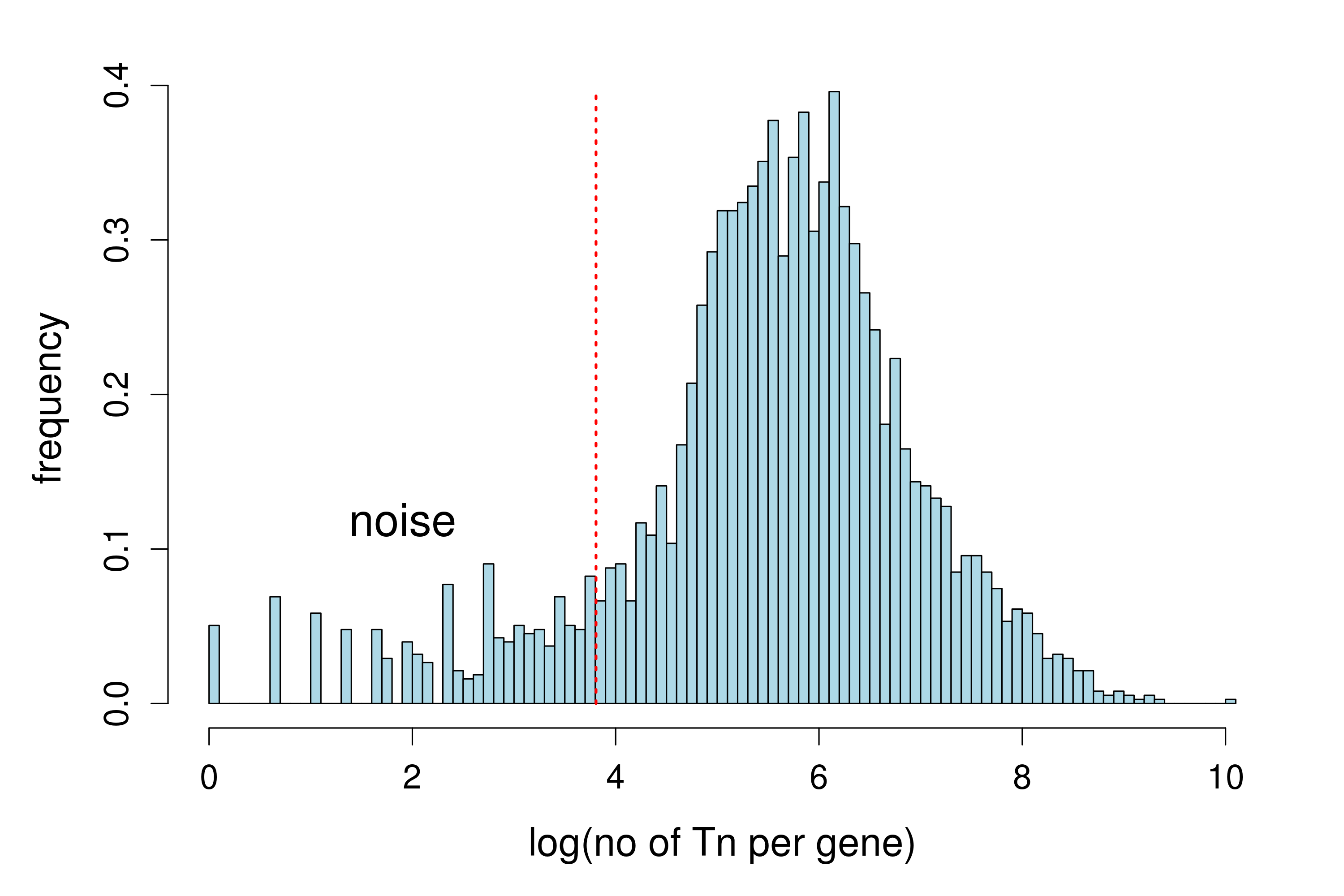


**Fig S2**. Noise trimming for the sample input2 (left) and the sample input3 (right). The horizontal axis represents the log of the number of transposon insertions per gene. The vertical axis stands for the frequency of the log of the number of transposon insertions per gene. The vertical dotted line represents the threshold. All genes with their insertion counts less than this threshold were treated as noise.


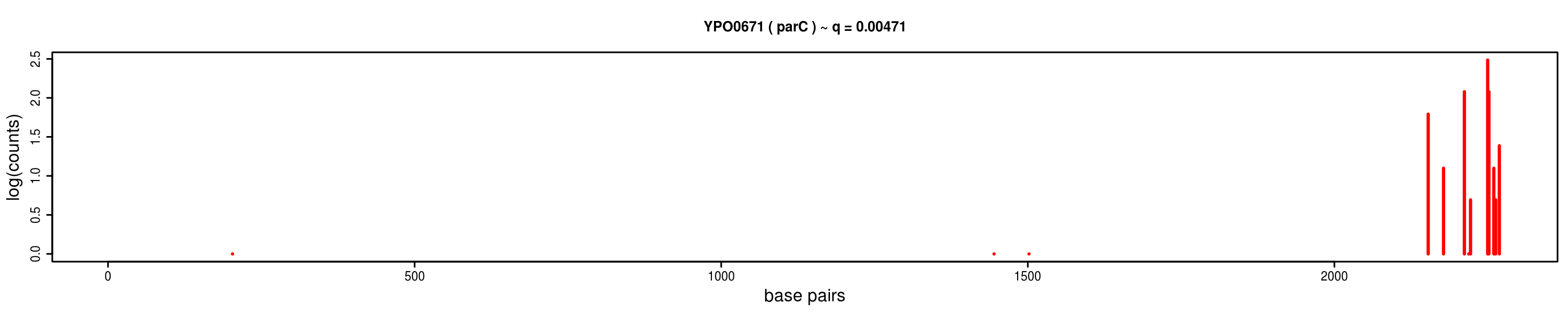


(A) A potential Type III essential gene


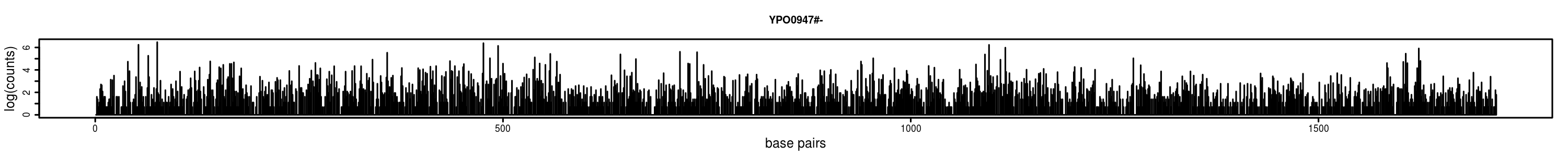


(B) A non-essential gene

**Fig S3**. An illustration of two transposon insertion patterns (A) This is a potential Type III essential gene because all insertions are located at the 3’ region (B) This is certainly a non-essential gene because the insertions are everywhere in the gene.


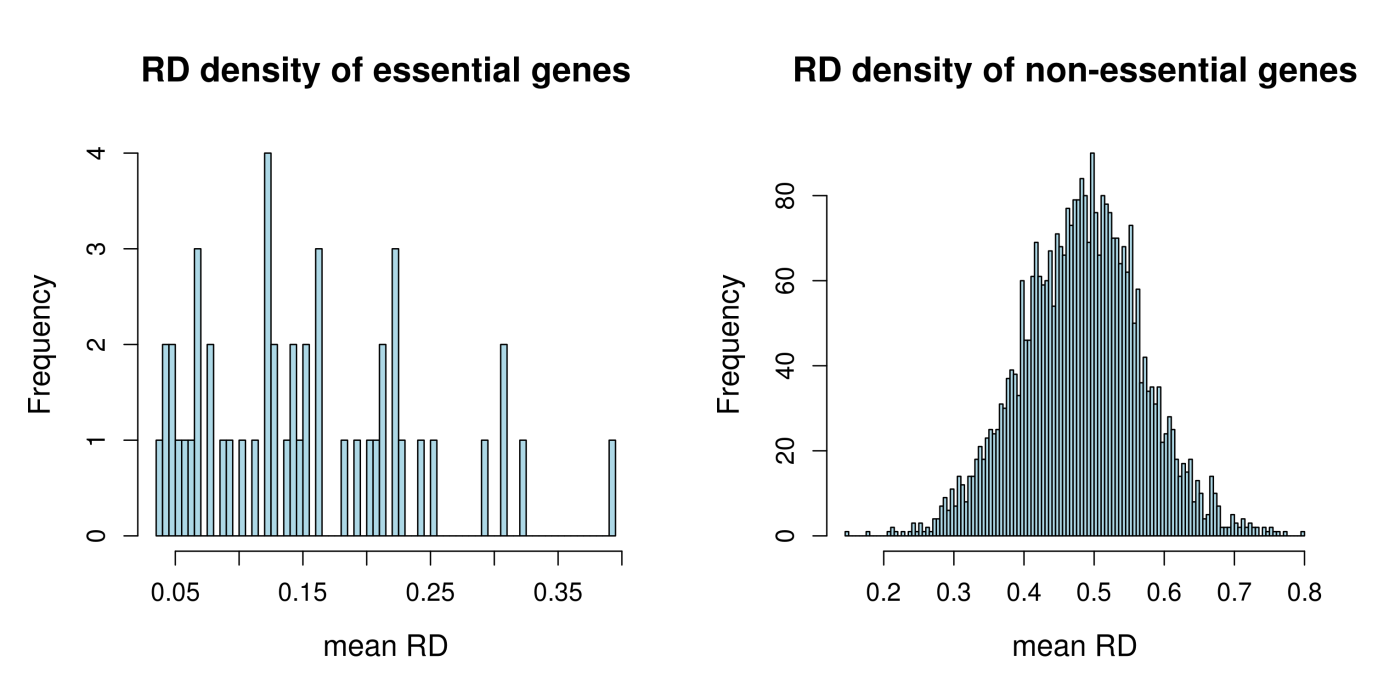


**Fig S4**. Mean RD densities for two categories of genes. The left panel shows the mean RD density of essential genes and the right panel shows the mean RD density of non-essential genes. The horizontal axis represents the log of mean RD values. The vertical axis stands for the frequency.


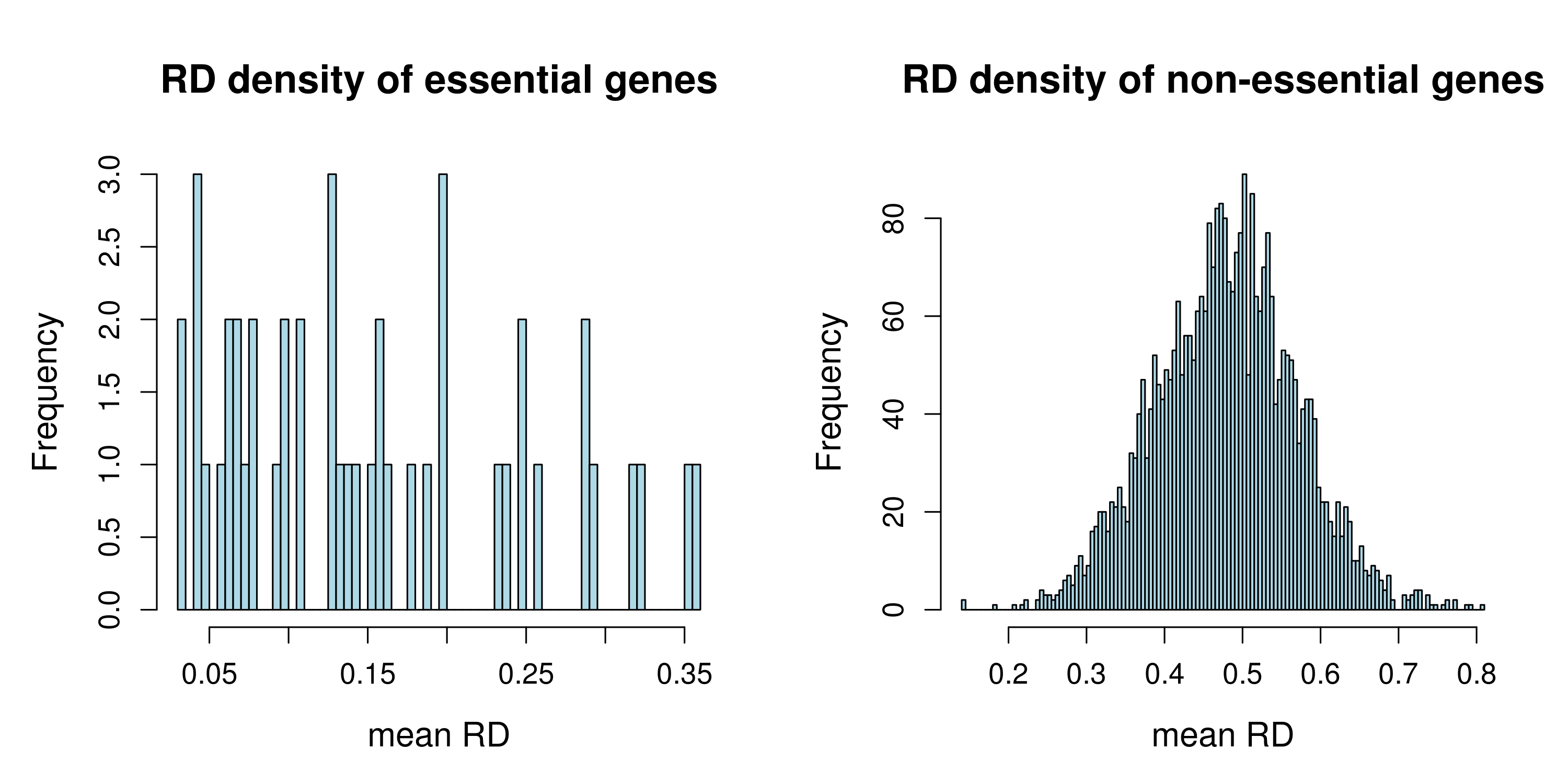


**Fig S5**. Distribution of RD values of essential genes (left) and non-essential genes (right) for the sample input2. The horizontal axis represents the log of mean RD values. The vertical axis stands for the frequency.


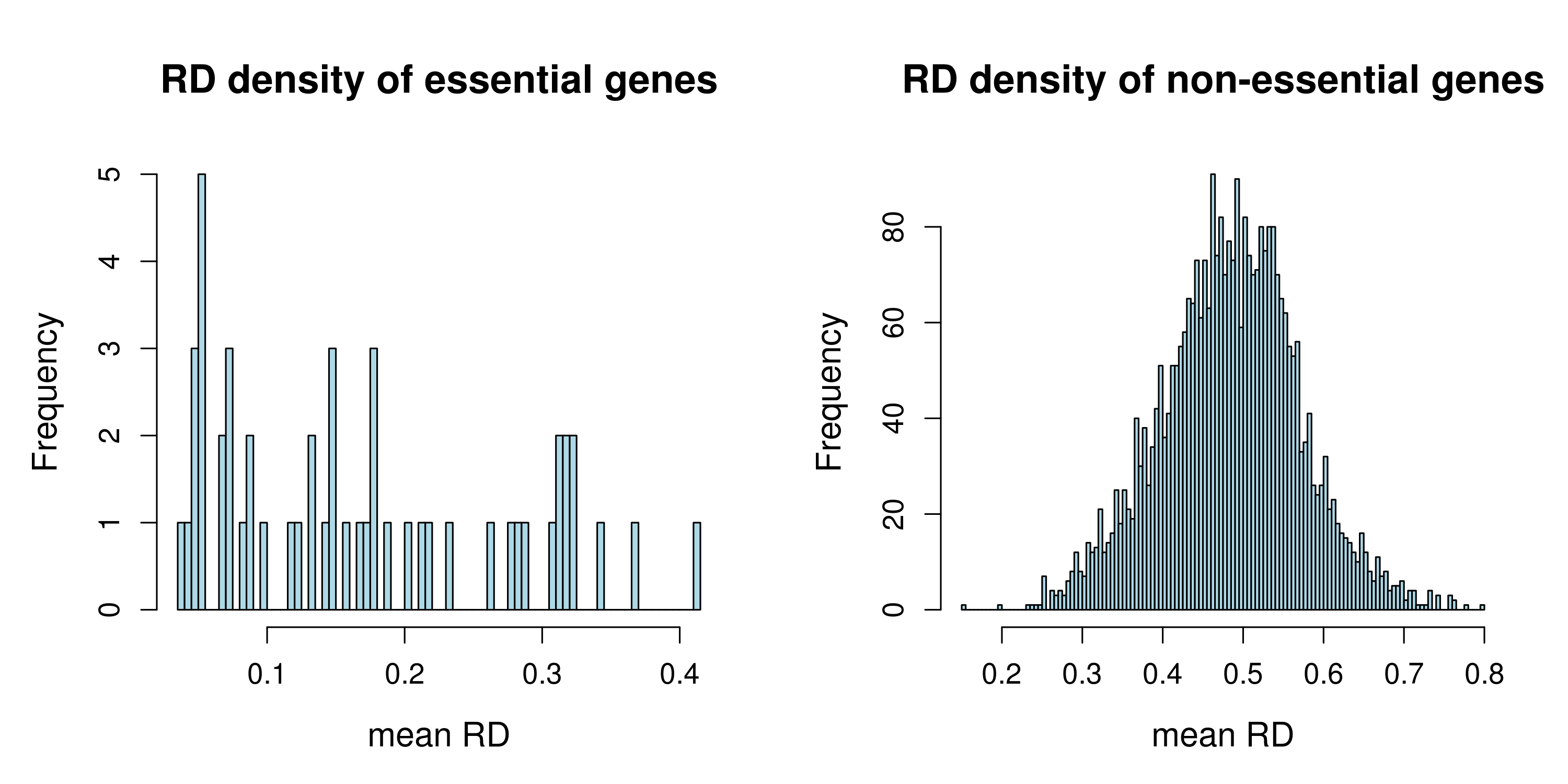


**Fig S6**. Distribution of RD values of essential genes (left) and non-essential genes (right) for the sample input3. The horizontal axis represents the log of mean RD values. The vertical axis stands for the frequency.


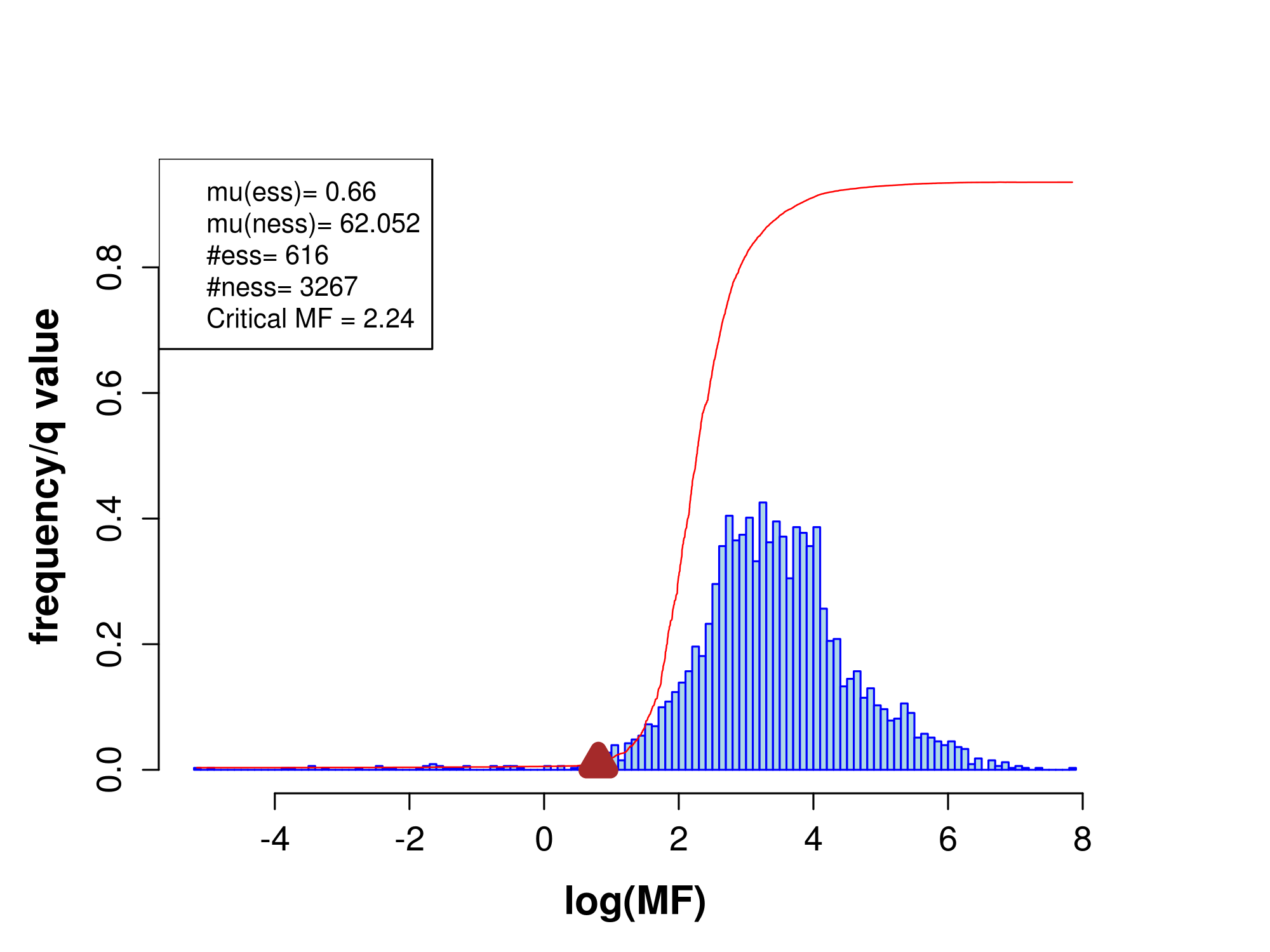

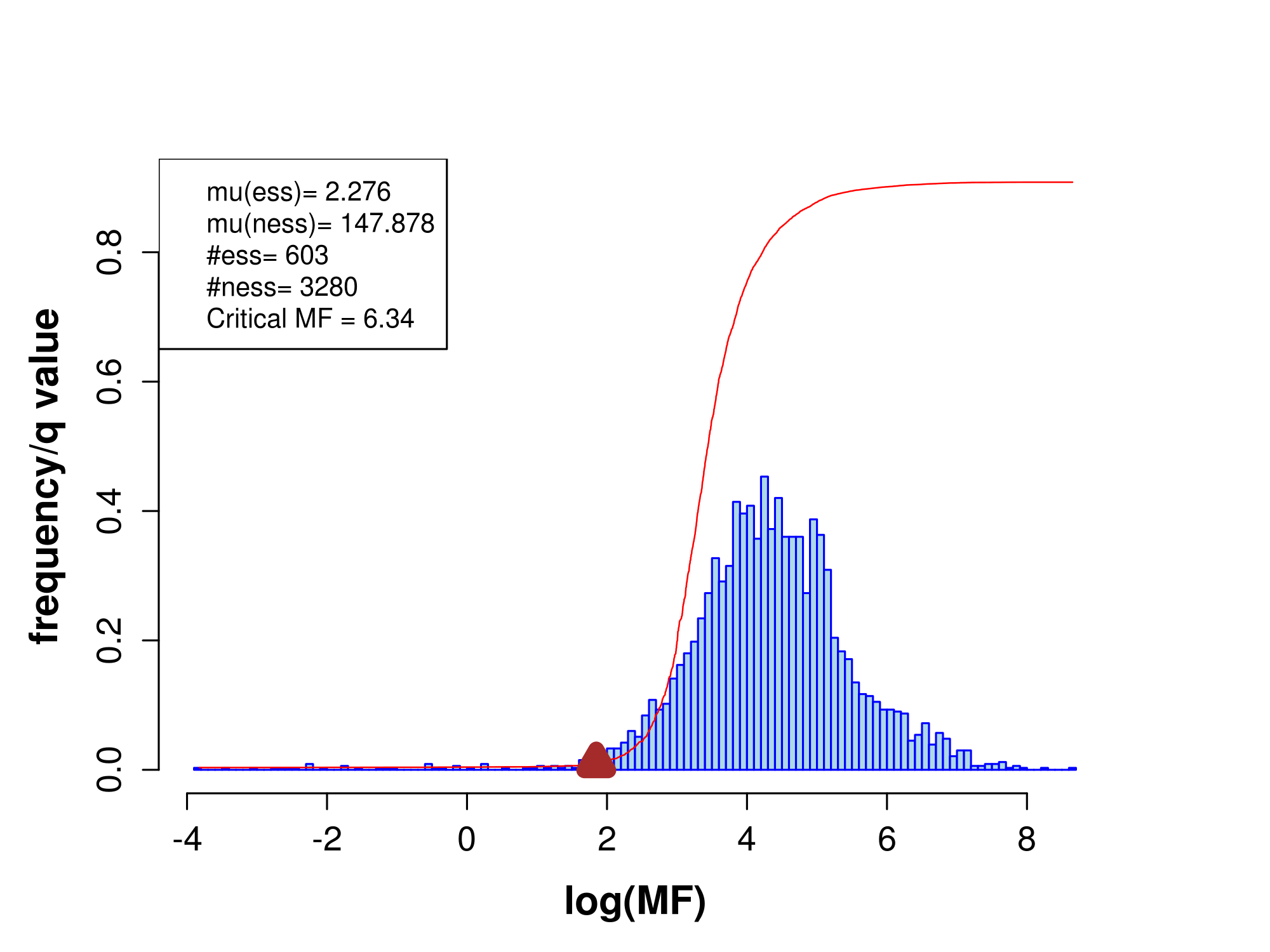


(A) (B)

**Fig. S7**. The prediction of essential genes for input2 (A) and input3 (B) using DEM. The curve represents the relationship between mutation feature values and the corresponding false discovery rates (*q* values). The triangle indicates the boundary separating between essential and non-essential genes. The horizontal axis represents the log of MF values. The vertical axis stands for the frequency and *q* values.


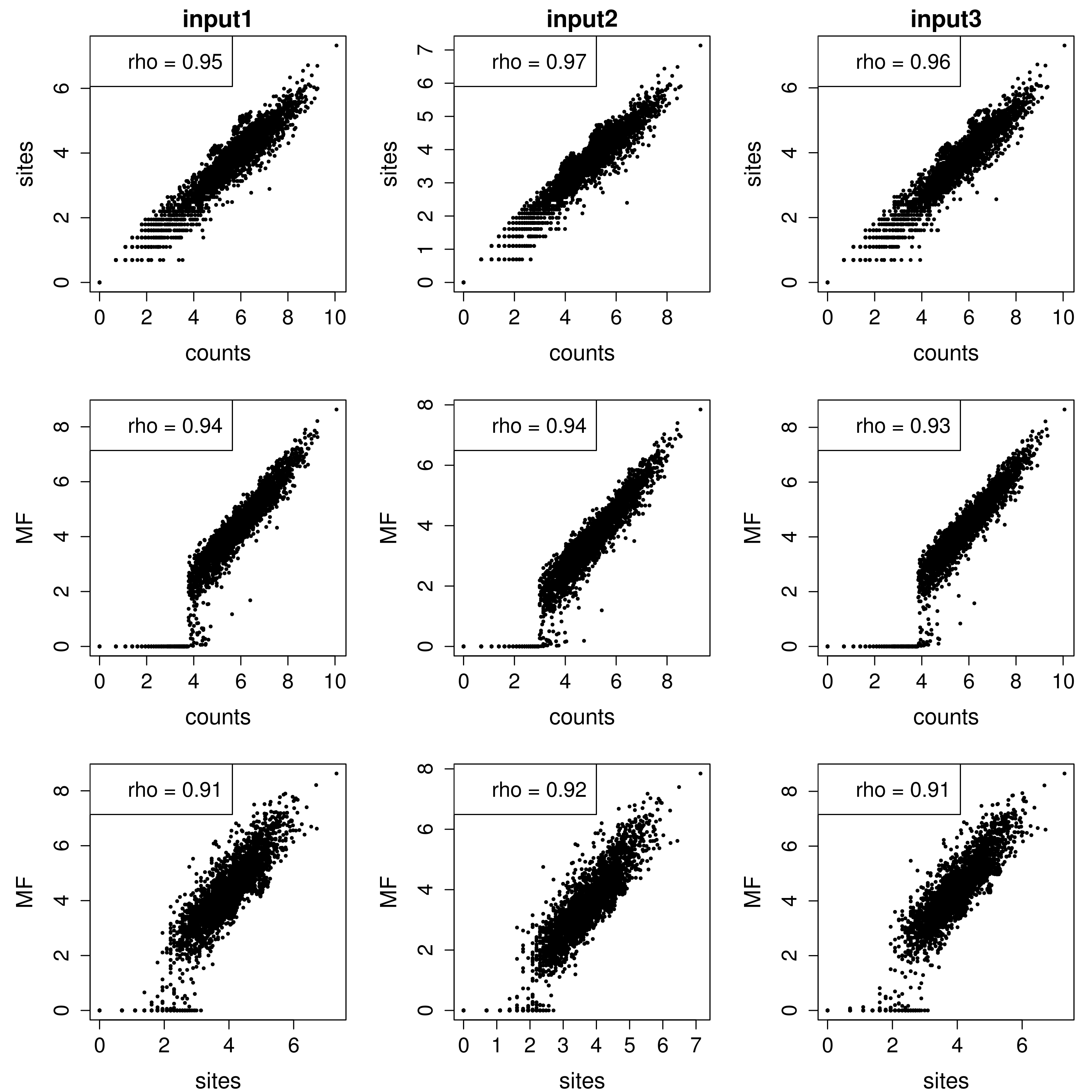


**Fig S8**. Correlation between three features (the count feature – transposon insertion counts per gene - , the site feature – transposon insertion sites per gene - and mutation feature). The first column is for input1. The second column is for input2 and the last column is for input3. rho stands for correlation coefficients.


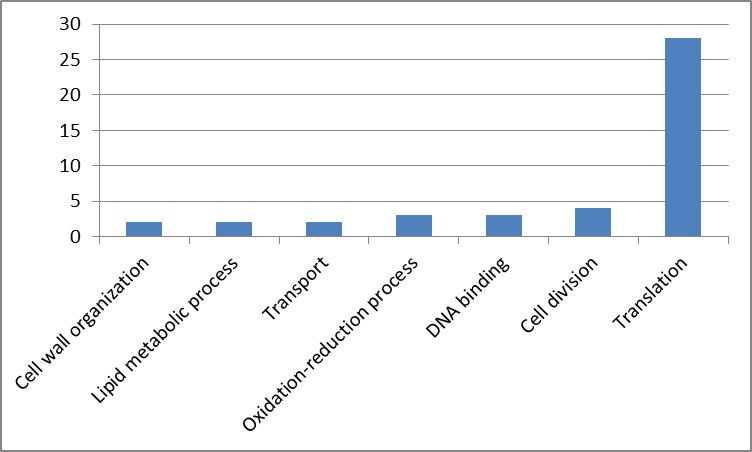


**Fig S9**: the functional analysis of Type I essential genes. Categories with only one gene were removed for visualisation.


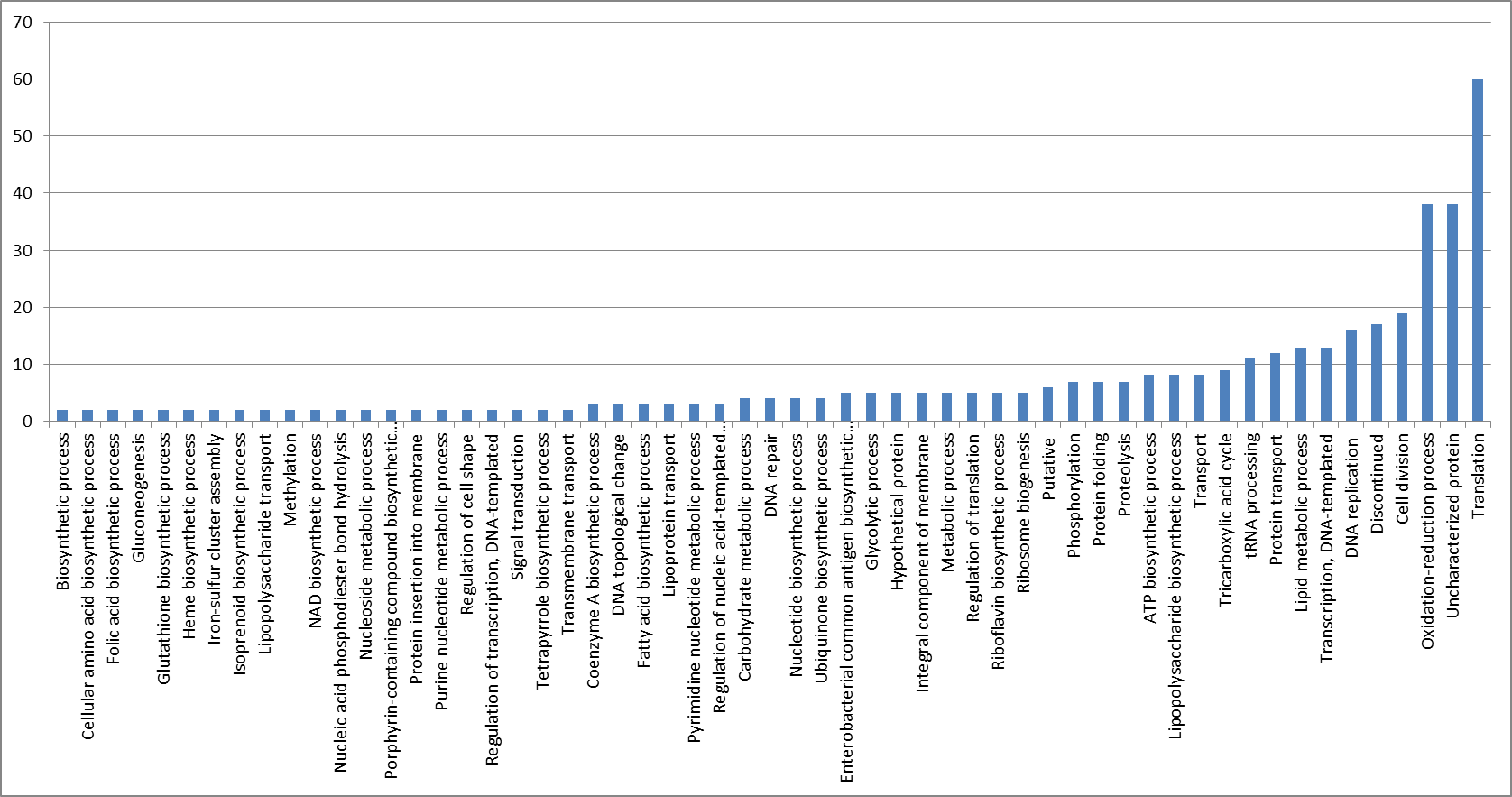


**Fig S10**: the functional analysis of Type II essential genes. Categories with only one gene were removed for visualisation.


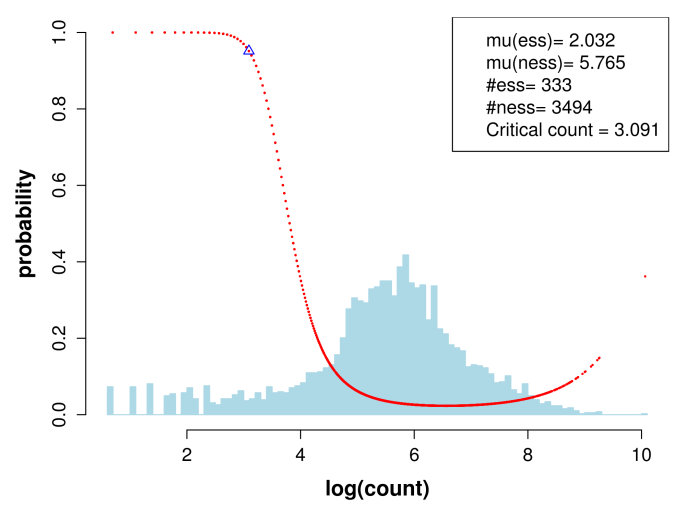

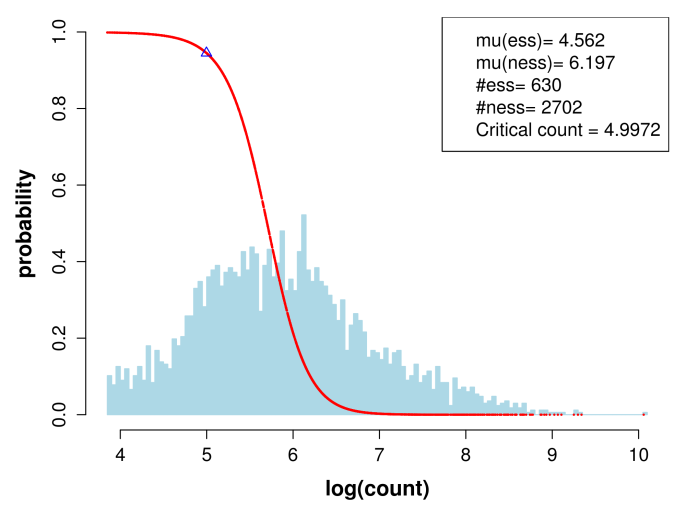


(A) (B)

**Fig S11**. The densities of the site feature values and the decision curve of TraDIS (posterior probability versus log (count)) for input1. (A) TraDIS built on non-noise-trimmed data. (B) TraDIS built on noise-trimmed data. The horizontal axes stand for log value of insertion counts and the vertical axes stand for the probability values.


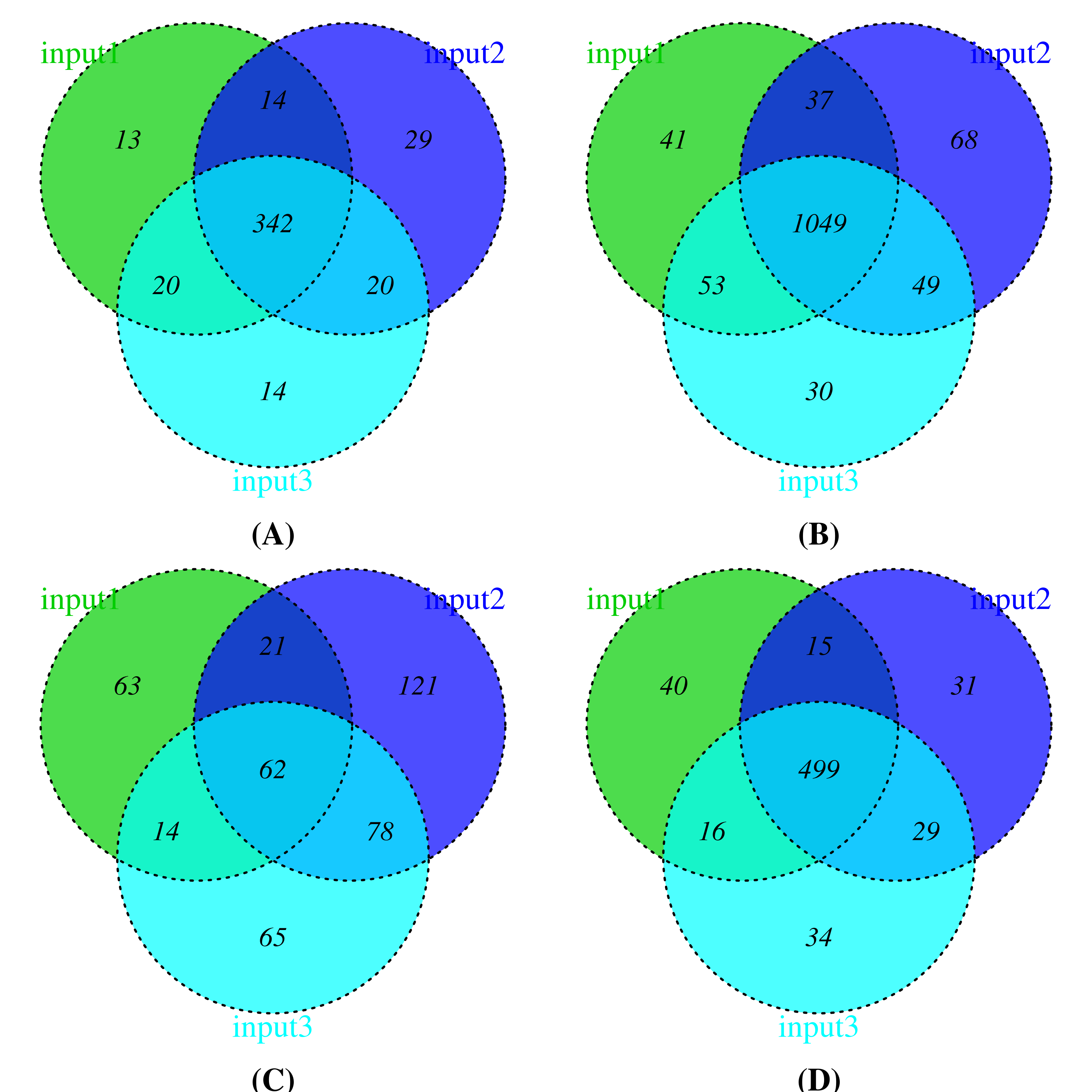


**Fig S12**. Venn diagrams of essential genes predicted by ESSENTIALS and TraDIS for three samples. (A) TraDIS predictions based on non-noise-trimmed data. (B) TraDIS predictions based on noise-trimmed data. (C) ESSENTIALS predictions based on non-noise-trimmed data. (D) ESSENTIALS predictions based on noise-trimmed data.


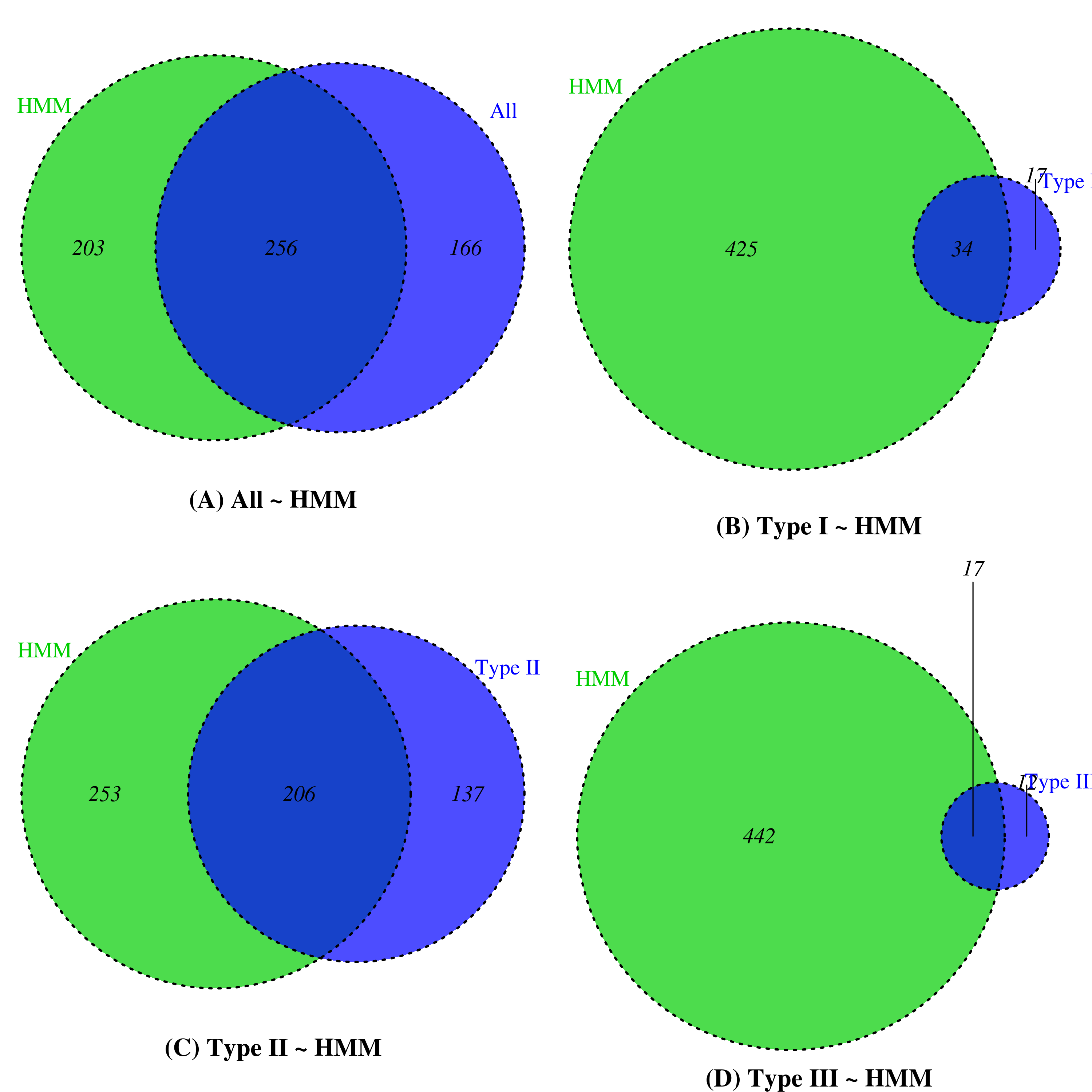


**Fig S13**: The comparison between out prediction and HMM prediction. (A) The comparison between all our prediction with HMM prediction. (B) The comparison between our Type I essential genes and HMM predicted essential genes. (C) The comparison between our Type II essential genes and HMM predicted essential genes. (D) The comparison between our Type III essential genes and HMM predicted essential genes. All comparisons are only based on genes which have gene symbols available.


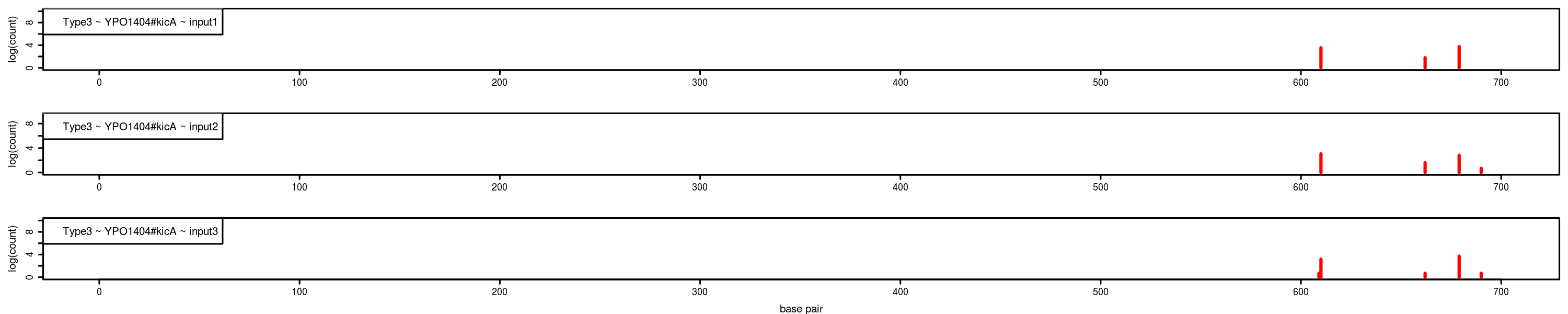


(A) gene kicA


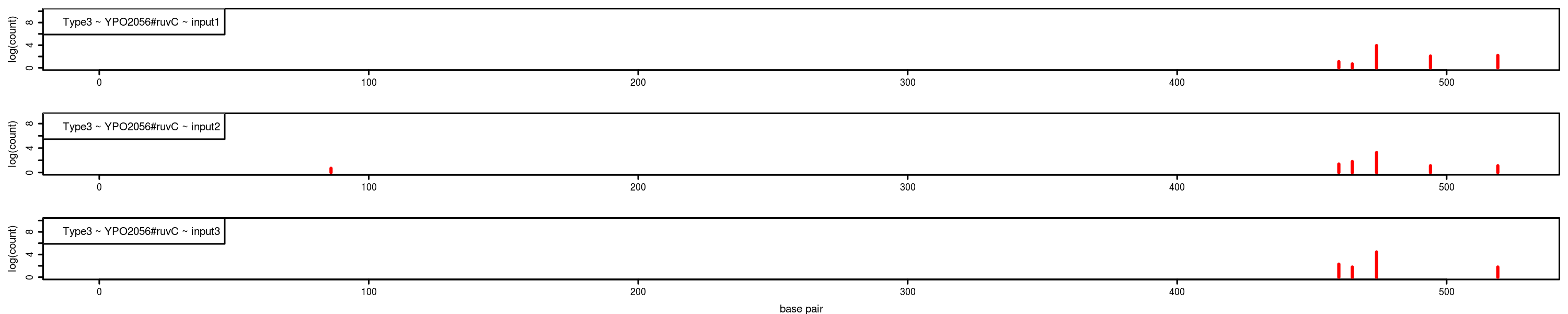


(B) gene ruvC


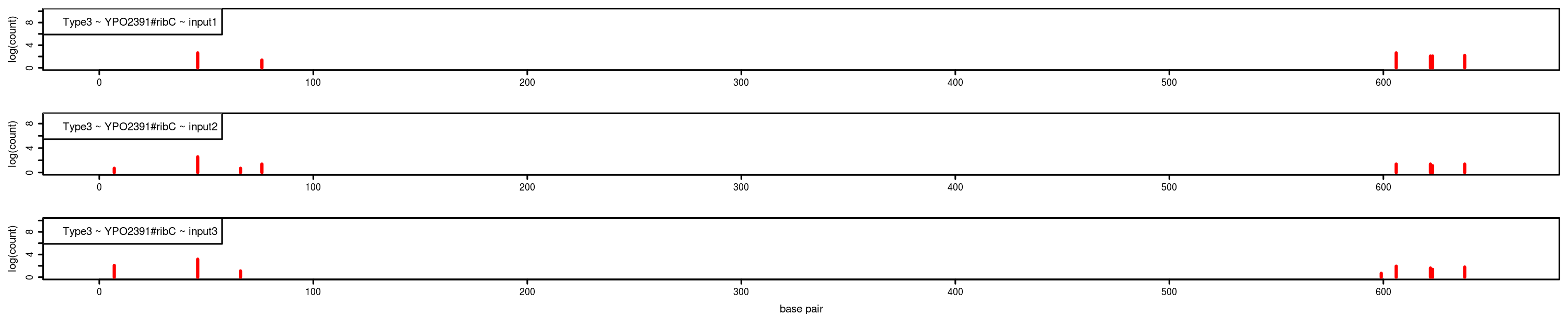


(C) gene ribC


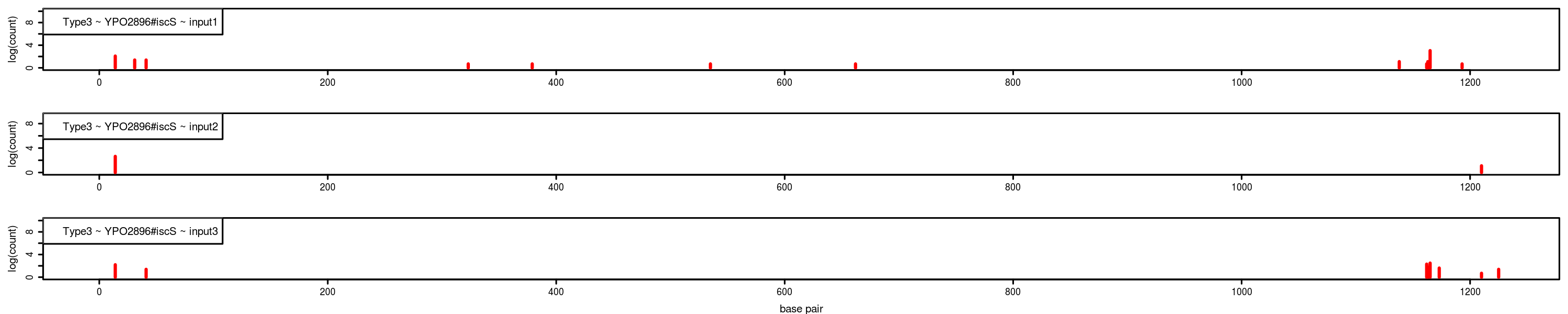


(D) gene iscS


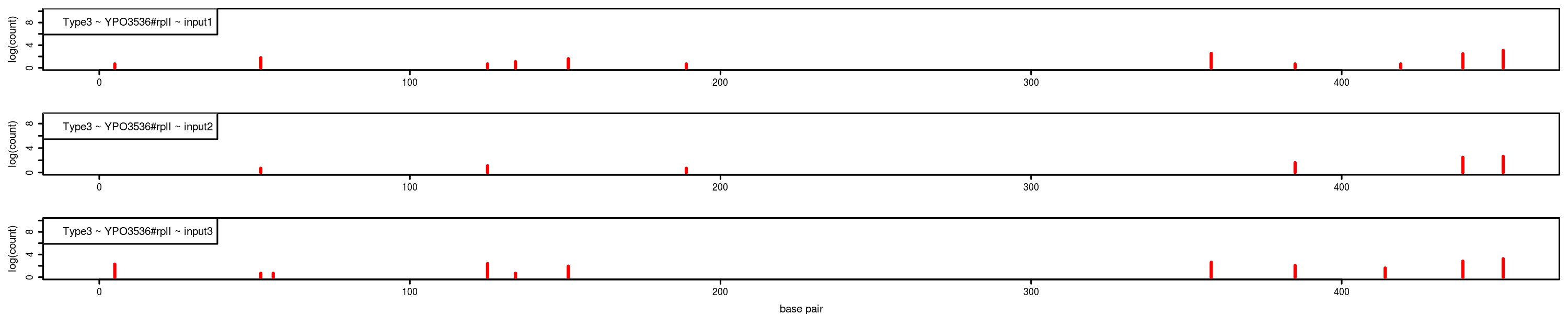


(E) gene rplI

**Fig S14**: 5 Type III essential genes predicted by our algorithm but missed in the HMM model.


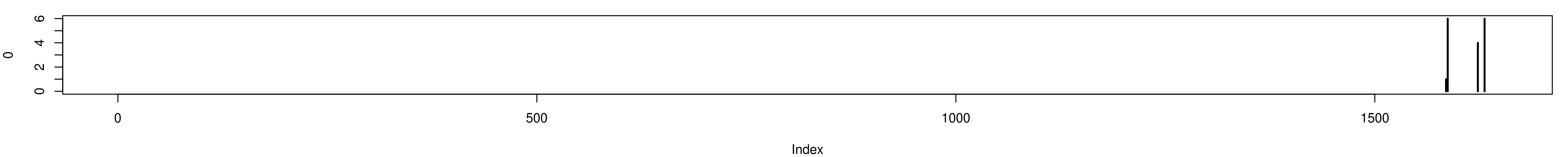


(A)


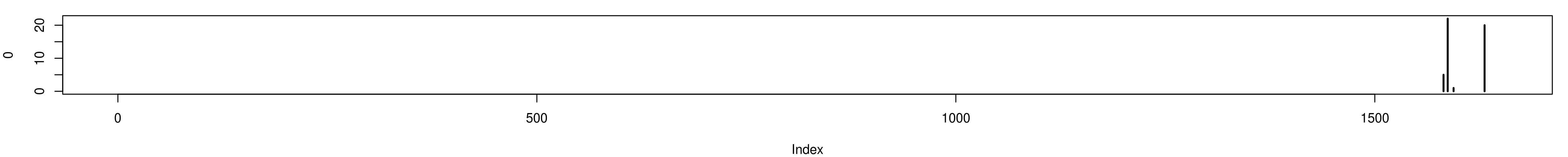


(B)

**Fig S15**. The transposon insertion pattern for the gene YPO3718 (pgi). (A) input2. (B) input3.


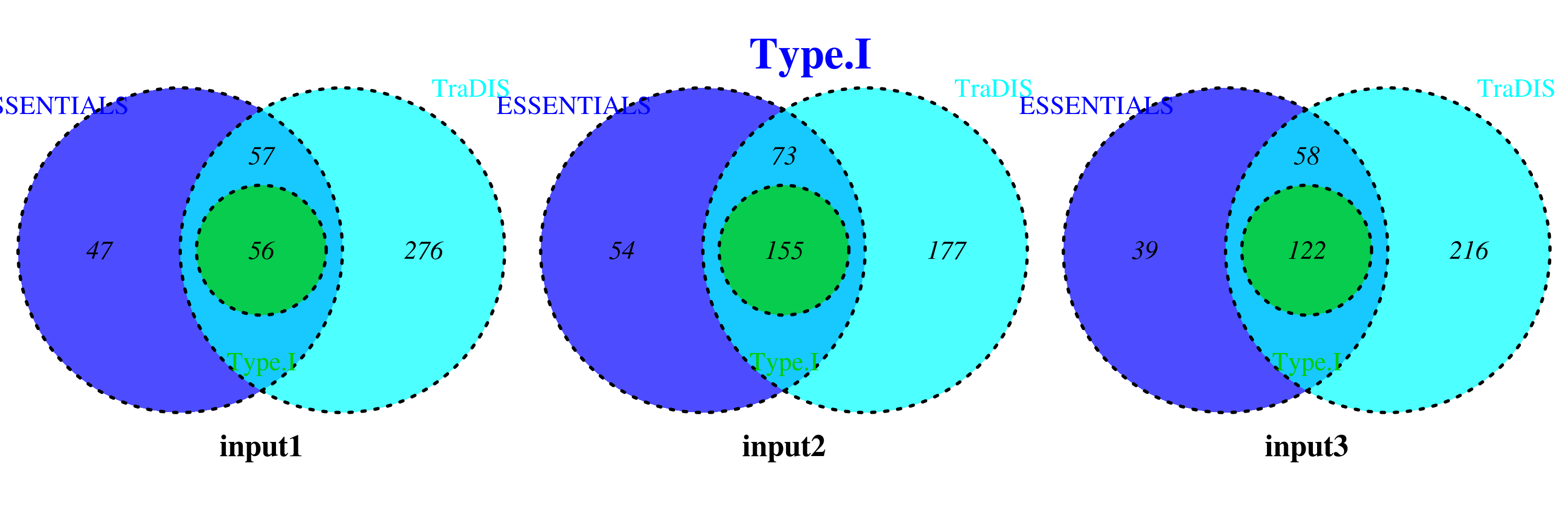


(A)


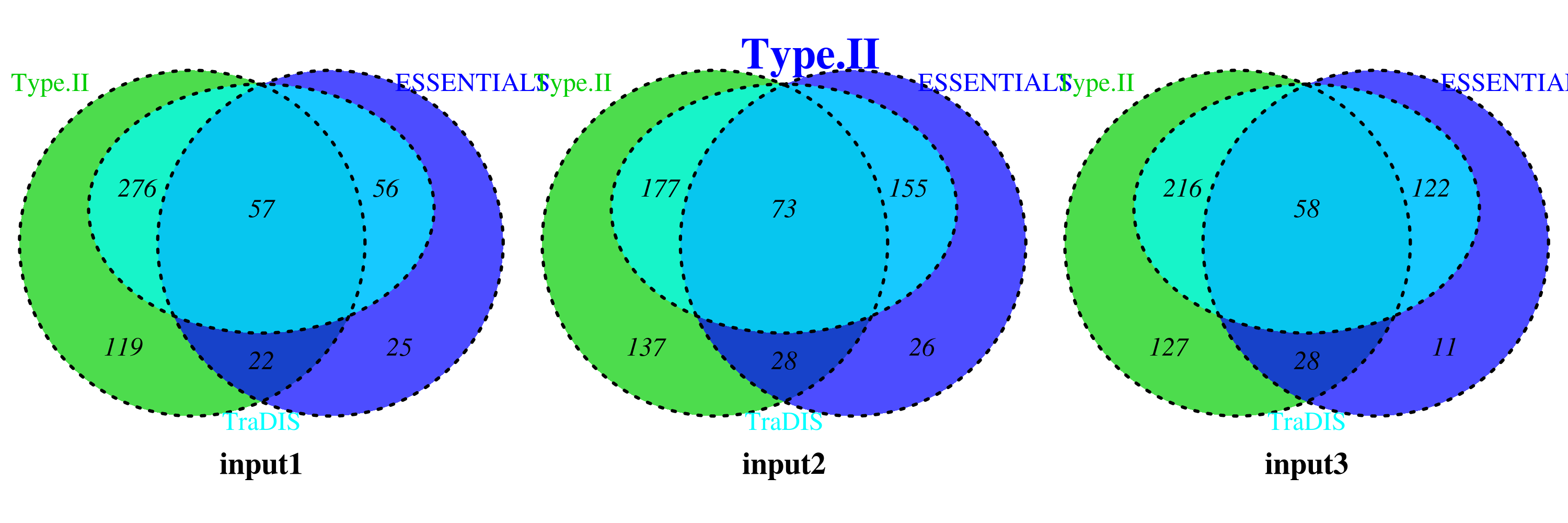


(B)


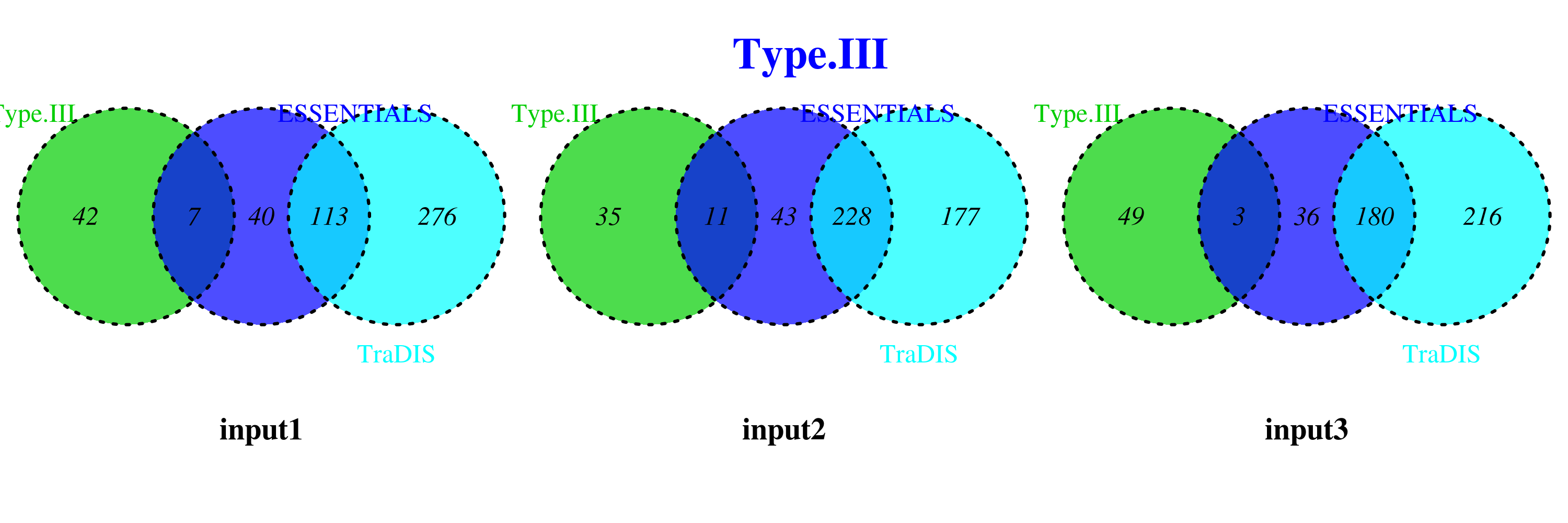


(C)

**Fig S16**. The relationship between the three types of essential genes and the predictions of ESSENTIALS and TraDIS. (A) Type I. (B) Type II. (C) Type III.

A B

C

D

**Fig S17**: Confirmation of putative essential targets: trans-complementation studies of (A). *murA* (B). *YPO3439* (C). *trmD* in liquid broth assays, and (D). *trmD* on solid media. Mutants were cultured under permissive (-■; 0.02% rhamnose) or non-permissive growth conditions (□; 0.1% glucose). Growth curves are representative of 2 separate experiments with 6 technical samples per value (mean+SEM)

**Table S1. Transposon data processed including raw sequencing reads, transposon sequences and mapped sequencing reads.**

| Sample | Raw | transposon insertion | Mapped |
| --- | --- | --- | --- |
| input1 | 20,806,077 | 16,479,505 | 3,534,184 |
| input2 | 14,616,979 | 11,973,458 | 1,673,302 |
| input3 | 21,801,223 | 17,304,679 | 3,839,915 |
| total | 57,224,279 | 45,757,642 | 9,047,401 |

**Table S2**. Transposon insertions at the distal end of genes. “Coverage” stands for the proportion of transposon insertions per base pair. “R3” stands for the proportion of insertions at 5% of the 3’ end genome-wise; “R5” stands for the proportion of insertions within 5% of the 5’ end genome-wise; “G3” stands for the number of genes which only have transposon insertions within 5% of the 3’ end; “G5” stands for the number of genes which only have transposon insertions within 5% of the 5’ end; “G3.5” stands for the number of genes which have transposon insertions within 5% of the 3’ and the 5’ end.

|  | Coverage | R3 | R5 | G3 | G5 | G3.5 |
| --- | --- | --- | --- | --- | --- | --- |
| input1 | 0.46 | 5.43 | 5.73 | 4 | 23 | 36 |
| input2 | 0.19 | 5.50 | 6.02 | 14 | 58 | 86 |
| input3 | 0.46 | 5.39 | 5.76 | 11 | 50 | 74 |

**Table S3**. Predicted essential genes using our approach. The total number of predicted essential genes includes Type I, II and II genes. The final column shows the *p* values of χ^2^ test.

|  | input1 | input2 | input3 | *p* (χ^2^ test) |
| --- | --- | --- | --- | --- |
| Type I essential genes | 56 | 155 | 122 | <0.001 |
| Type II essential genes | 474 | 415 | 429 | 0.12 |
| Type III essential genes | 49 | 46 | 52 | 0.83 |
| Total predicted essential genes | 579 | 616 | 603 | 0.56 |
| % of Type I essential genes | 9.7 | 25.2 | 20.2 |  |
| % of Type II essential genes | 81.8 | 67.3 | 71.2 |  |
| % of Type III essential genes | 8.5 | 7.5 | 8.6 |  |

**Table S4**. Kolmogorov–Smirnov test of essential genes in genomes.

| Sample | Type I | Type II | Type III |
| --- | --- | --- | --- |
| input1 | 0.020236 | 0.027903 | 0.856115 |
| input2 | 0.002703 | 0.009757 | 0.828765 |
| input3 | 0.000153 | 0.001733 | 0.73373 |

**Table S5.** The analysis of genes with insertions only at distal regions using our algorithm. “II” stands for Type II essential genes. “III” stands for Type III essential genes. The distal regions were within 5% of the distal ends.

|  | 3' (II and III) | 5' (II and III) | 3' and 5' (II and III) | 3' (III) | 5' (III) | 3' and 5' (III) |
| --- | --- | --- | --- | --- | --- | --- |
| input1 | 4 | 23 | 36 | 0 | 0 | 2 |
| input2 | 14 | 58 | 86 | 0 | 2 | 5 |
| input3 | 11 | 50 | 74 | 0 | 4 | 5 |

**Table S6.** The analysis of genes with insertions only at distal regions using ESSENTIALS and TraDIS. The distal regions were within 5% of the distal ends.

|  | TraDIS | | | ESSENTIALS | | |
| --- | --- | --- | --- | --- | --- | --- |
|  | 3' | 5' | 3' and 5' | 3' | 5' | 3' and 5' |
| input1 | 4 | 23 | 31 | 0 | 3 | 5 |
| input2 | 14 | 49 | 67 | 2 | 17 | 24 |
| input3 | 11 | 40 | 60 | 4 | 7 | 13 |

**Table S7.** Bacterial strains and culture conditions. *Y. pestis* strains were cultured in blood agar base (BAB) broth or BAB agar supplemented with hemin (0.025%) at 28^o^C. Strains of *E. coli* were cultured in Luria-Bertani (LB) broth. When required media was supplemented with kanamycin (25µg ml^-1^), trimethoprim (100µg ml^-1^) and chloramphenicol (25µg ml^-1^). L-rhamnose (0.02%) and L-glucose (0.1%) were added as appropriate for the validation of targets.

| Strain or plasmid Strains | Description | Source or reference |
| --- | --- | --- |
| *E.coli* JM109 | Cloning strain *end*A1, *rec*A1, *gyr*A96, *thi*, *hsd*R17 (r_k_^-^ m_k_^+^), *rel*A1, *sup*E44, Δ (*lac-pro*AB), [F’ *tra*D36, *pro*AB, *lac*IqZΔM15]. | Promega |
| *Y. pestis* CO92 | Sequenced strain, Biovar Orientalis, fully virulent | Ref ^1^ |
| *Y. pestis* /pAJD434 | *Y. pestis* CO92 containing pAJD434 | This study |
| *Y. pestis* /pAJD434/pBADrha-*fbaA* | *Y. pestis* CO92 containing pAJD434 and pBADrha-*fbaA* | This study |
| *Y. pestis* /pAJD434/pBADrha-*murA* | *Y. pestis* CO92 containing pAJD434 and pBADrha-*murA* | This study |
| *Y. pestis* /pAJD434/pBADrha-*accA* | *Y. pestis* CO92 containing pAJD434 and pBADrha-*accA* | This study |
| *Y. pestis* /pAJD434/pBADrha-*yidC* | *Y. pestis* CO92 containing pAJD434 and pBADrha-*yidC* | This study |
| *Y. pestis* /pAJD434/pBADrha-*YPO3439* | *Y. pestis* CO92 containing pAJD434 and pBADrha-*YPO3439* | This study |
| *Y. pestis* /pAJD434/pBADrha-*trmD* | *Y. pestis* CO92 containing pAJD434 and pBADrha-*trmD* | This study |
| *Y. pestis* /pAJD434/pBADrha-*ispG* | *Y. pestis* CO92 containing pAJD434 and pBADrha-*ispG* | This study |
| *Y. pestis/pAJD434/pBADrha-spoT* | *Y. pestis* CO92 containing pAJD434 and pBADrha-*spoT* | This study |
| *Y. pestis* Δ*fbaA* | *Y. pestis* CO92 in which the chromosomal *fbaA* gene has been replaced by a kanamycin cassette, cured of plasmid pAJD434 by heat shock | This study |
| *Y. pestis* Δ*murA* | *Y. pestis* CO92 in which the chromosomal *murA* gene has been replaced by a kanamycin cassette, cured of plasmid pAJD434 by heat shock | This study |
| *Y. pestis* Δ*accA* | *Y. pestis* CO92 in which the chromosomal *accA* gene has been replaced by a kanamycin cassette, cured of plasmid pAJD434 by heat shock | This study |
| *Y. pestis* Δ*yidC* | *Y. pestis* CO92 in which the chromosomal *yidC* gene has been replaced by a kanamycin cassette, cured of plasmid pAJD434 by heat shock | This study |
| *Y. pestis* Δ*ispGA* | *Y. pestis* CO92 in which the chromosomal *ispG* gene has been replaced by a kanamycin cassette, cured of plasmid pAJD434 by heat shock | This study |
| *Y. pestis* Δ*YPO3439* | *Y. pestis* CO92 in which the chromosomal gene *YPO3439* has been replaced by a kanamycin cassette, cured of plasmid pAJD434 by heat shock | This study |
| *Y. pestis* Δ*trmD* | *Y. pestis* CO92 in which the chromosomal *trmD* gene has been replaced by a kanamycin cassette, cured of plasmid pAJD434 by heat shock | This study |
| *Y. pestis ∆spoT* | *Y. pestis* CO92 in which the chromosomal *spoT* gene has been replaced by a kanamycin cassette, cured of plasmid pAJD434 by heat shock | This study |
| Plasmids |  |  |
| pBADrha | Expression vector containing the rhamnose inducible promoter P_rhaB_, *rhaR* and *rhaS.* Ori p15*,* Cat^R^ | Ref ^2^ |
| pBADrha-*murA* | pBADrha containing the *murA* gene of Y. pestis CO92 | This study |
| pBADrha-*accA* | pBADrha containing the *accA* gene of Y. pestis CO92 | This study |
| pBADrha-*yidC* | pBADrha containing the *yidC* gene of Y. pestis CO92 | This study |
| pBADrha-*fbaA* | pBADrha containing the *fbaA* gene of Y. pestis CO92 | This study |
| pBADrha-*YPO3439* | pBADrha containing the gene *YPO3439* of Y. pestis CO92 | This study |
| pBADrha-*trmD* | pBADrha containing the *trmD* gene of Y. pestis CO92 | This study |
| pBADrha-*ispG* | pBADrha containing the *ispG* gene of Y. pestis CO92 | This study |
| pBADrha-*spoT* | pBADrha containing the *spoT* gene of Y. pestis CO92 | This study |
| pK2 | pGEM-T-Easy vector with Kan^R^ gene insertion at the *Bgl* II restriction site | Ref ^3^ |
| pAJD434 | Arabinose inducible λ red recombinase genes, Tp^R^ | Ref ^4^ |

**Table S8**. Sequences of adapters used in this study and primer sequences used during preparation of mutant libraries for sequencing.

| **Adapter** | **Sequence** | **Comment** |
| --- | --- | --- |
| **Ind_Ad_T** | ACACTCTTTCCCTACACGACGCTCTTCCGATC*T | *Phosphorothioate |
| **Ind_Ad_B** | pGATCGGAAGAGCGGTTCAGCAGGAATGCCGAGACCGATCTC | Phosphorylated |
| **Primer** | **Sequence** | **Comment** |
| **PE_PCR_V3.3** | **CAAGCAGAAGACGGCATACGA**GATCGGTACACTCTTTCCCTACACGACGCTCTTCCGATCT | Flow cell binding region in bold |
| **Yp EZ_Tn PCR** | **AATGATACGGCGACCACCGAGATCTACAC**ACCTACAACAAAGCTCTCATCAACC | Flow cell binding region in bold |
| **Yp EZ_Tn seq** | TGCAAGCTTCAGGGTTGAGA |  |

**Table S9. Oligonucleotides used in this study. Restriction endonuclease recognition sites required for cloning purposes are underlined.**

| Name | Sequence | Description |
| --- | --- | --- |
| accA F | GGAATTCCATATGAGTCTGAATTTTCTT | Complementation of *accA* ORF in pBADrha |
| accA R | TGCTAGTCTAGATCAGCAATAGCCGTAG | Complementation of *accA* ORF in pBADrha |
| fbaA F | GGAATTCCATATGTCTAAAATTTTTGA | Complementation of *fbaA* ORF in pBADrha |
| fbaA R | TGCTAGTCTAGATTACAGTACGTCGATG | Complementation of *fbaA* ORF in pBADrha |
| ispG F | GCTTACCATATGCATAACGGATCCCCTATTATTCG | Complementation of *ispG* ORF in pBADrha |
| ispG R | TGACATCTAGACTATTTATTATCATCCAATTGG | Complementation of *ispG* ORF in pBADrha |
| murA F | GGAATTCCATATGGATAAGTTTCGTGTGC | Complementation of *murA* ORF in pBADrha |
| murA R | GGATCCTTACTCGCCTTTCACGC | Complementation of *murA* ORF in pBADrha |
| spoT F | CATATGTACCTGTTTGAAAGCCTGAA | Complementation of *spoT* ORF in pBADrha |
| spoT R | TCTAGATTAATTGCGATTACGGCTAAC | Complementation of *spoT* ORF in pBADrha |
| trmD F | ACGTGCATTAATGCGATAGCGAGTGGAACAAA | Complementation of *trmD* ORF in pBADrha |
| trmD R | AAAACTGCAGTCAGGGCTTATGTTCCCGTT | Complementation of *trmD* ORF in pBADrha |
| yidC F | GCTTAGCATATGGATTCGCAACGCAATC | Complementation of *yidC* ORF in pBADrha |
| yidC R | TGACGTCTAGATTATTTTTTCTTCTCGCGGC | Complementation of *yidC* ORF in pBADrha |
| YPO3439 F | CATATGTTTGGTGTATTAGACCGCTA | Complementation of YPO3439 ORF in pBADrha |
| YPO3439 R | TCTAGATTACCGCCGTTTTAGCAGCA | Complementation of YPO3439 ORF in pBADrha |
| accA H1 | TGGTAGGTAATGAGCAAGTGGAACTGGAATTTGACTAAAATAGGAATGCTATGAGCCATATTCAACGGG | accA-kan PCR product for λ red mutagenesis |
| accA H2 | AAAAACCGGCGCTTAAATTCCGCACCGGCTTTTATCAGTTGGCAATCAACTTAGAAAAACTCATCGAGCATC | accA-kan PCR product for λ red mutagenesis |
| fbaA H1 | CAGCGAACCTATTCACATTTATCTTCGGCCGACGATACAGGACAACTTACATGAGCCATATTCAACGGG | fbaA-kan PCR product for λ red mutagenesis |
| fbaA H2 | AACCTCAAAGGCCCCGTAGGGCCTTTTAGGTCAGTCCGAACAGACTGGAATTAGAAAAACTCATCGAGCATC | fbaA-kan PCR product for λ red mutagenesis |
| ispG knockoutF | TGACAAATATCATGTTGTAAGAACTACACGACCGTAAAGGAGAGTATGTAATGAGCCATATTCAACGGG | ispG-kan PCR product for λ red mutagenesis |
| ispG knockoutR | GCATAACTGTCTGTTTGATTCTGGCCGACAACAAGGATTGCCGATAGAGCTTAGAAAAACTCATCGAGCATC | ispG-kan PCR product for λ red mutagenesis |
| murA H1 | GCGAATTCGAATTTGACAACAAGATTTGACAACAACCAAGAGTGGTCACAATGAGCCATATTCAACGGG | murA-kan PCR product for λ red mutagenesis |
| murA H2 | ACCGAACACGATCCGCTTTTTAGCGATCAGCTTTCTGTCGATCTGCGGATTTAGAAAAACTCATCGAGCATC | murA-kan PCR product for λ red mutagenesis |
| spoT H1 | GGTTACCGCCATTGCTGAAGGTCGTCGTTAATTAGACTGCGAGTCTGCCTATGAGCCATATTCAACGGG | spoT-kan PCR product for λ red mutagenesis |
| spoT H2 | GGTGGCAAGCATGTCACAAATCCGCGCATAGCGTTGAGGATTCATAGGCGTTAGAAAAACTCATCGAGCATC | spoT-kan PCR product for λ red mutagenesis |
| trmD H1 | AACGTGTTGAAGTAGATTGGGATCCTGGTTTTTGACCTCCGAATTAAACGACAAGGGGTGTTATGAGCC | trmD-kan PCR product for λ red mutagenesis |
| trmD H2 | TATCAGGACCATTTGCGCGCGGCACAATGTTCCCTTCATAGTTCTGTTGCTTAGAAAAACTCATCGAGCATC | trmD-kan PCR product for λ red mutagenesis |
| yidC knockoutF | GGTGATGACCCCGTGCCGCCGAAACTCGACGATAACAGAGAACACTAACGATGAGCCATATTCAACGGG | yidC-kan PCR product for λ red mutagenesis |
| yidC knockoutR | ATAAGGCGGTCATATTGACCGCCCTAAATACTCATGATTATCGCTGTGGGTTAGAAAAACTCATCGAGCATC | yidC-kan PCR product for λ red mutagenesis |
| YPO3439 H1 | GATGGCCTGATGGCTCGTAAATTACGTGCTCGATTGAGAGGTGCTGCCTGATGAGCCATATTCAACGGG | YPO3439-kan PCR product for λ red mutagenesis |
| YPO3439 H2 | ATGCAACTGTTATTCCACTACGTTTAGTCTAAGTGCTGAAAAAAACGTCATTAGAAAAACTCATCGAGCATC | YPO3439-kan PCR product for λ red mutagenesis |
| accA check F | GCGAACGTTGGTAGGTAATG | Screening primer |
| accA check R | AATTGCCAGCACGTATCCTC | Screening primer |
| fbaA check F | CGCGCTAAGCAGTAATTTGG | Screening primer |
| fbaA check R | CCAGGCCATTAAGTCAGTGATGACAG | Screening primer |
| ispG  screenF | GAACTACACGACCGTAAAGG | Screening primer |
| ispG  screenR | GTTTGATTCTGGCCGACAAC | Screening primer |
| murA check F | CGGGATCGCAAACTAAATGG | Screening primer |
| murA check R | TGGGTACCTTGACGCCGATG | Screening primer |
| spoT check F | TGGACTTGCTCCAGACAGAC | Screening primer |
| spoT check R | CGATGTTCGTGAGCGCCAAG | Screening primer |
| trmD check F | CACTGCTCAACGTGTTGAAG | Screening primer |
| trmD check R | CGGAATGCAGGTACATCTTG | Screening primer |
| yidC  screenF | CCGAAACTCGACGATAACAG | Screening primer |
| yidC  screenR | TGACCGCCCTAAATACTC | Screening primer |
| YPO3439 check F | TTGAGTCAACTGCGTCTACC | Screening primer |
| YPO3439 check R | TGGATGTGGGCTGTTAATGG | Screening primer |
| lcrVF | TCTACCCGAGGATGCCATTC | Screening primer for pCD1 |
| lcrVR | TCTAGCAGACGTTGCATCAC | Screening primer for pCD1 |
| gamF | TGGGAATTCGAGCTCTAAGG | Screening primer for λ red helper plasmid pAJD434 |
| gamR | TGCGAGTGCAGTACTCATTC | Screening primer for λ red helper plasmid pAJD434 |

**The distal effect model**

**Introduction**

All the expressions of insertions in this document refer to the insertion of a transposon into a genome. Classifying genes into non-mutational (essential) and mutational (non-essential) based on transposon-sequencing technology data is an *unsupervised* learning process. This process aims to find a mapping function from a genotype variable to a phenotype variable. The classification (or free classification or clustering) is based on such an established mapping function. In the context of this paper, a genotype variable represents a mutation feature describing the genetic reason of mutation and a phenotype variable stands for mutation status describing whether a gene is mutational or non-mutational. We denote a genotype variable by *X* and a phenotype variable by *T*. The mapping function between them is defined as

| $f(X)\mapsto T$ | (S1) |
| --- | --- |

where $f(X)$ is a mapping function and means "maps to". For an individual gene, we have $f(x_{i})\mapsto t_{i}$, where $x_{i}$ is the mutation feature value of the *i*^th^ gene and $t_{i}$ is the mutation status value of the *i*^th^ gene. In addition to estimating such a mapping function, the challenge is the data complexity. It roots from the fact that explicit definition of a mutation feature is normally unavailable when a new transposon-sequencing data set arrives. Each gene may attract insertion sites from zero to many. An individual site may attract insertions from one to many depending on the coverage depth of sequencing as well as the genetic property of a gene. The number of insertions at the same site is called insertion count or simply count. The significance of mutation of a gene should depend on where an insertion is and how insertion distributes in a gene. Without the negative selection, the null hypothesis is that a transposon may be inserted into a genome randomly with a uniform distribution across genes and across base pairs. However, due to the negative selection, different genes will have different insertion sites and different insertion counts. The key question is how to integrate this two-dimensional information (site and count) into a single genotype variable by which a mapping function between a genotype variable and a phenotype variable can be estimated.

**Relative distance to the distal ends**

We denote a site vector of the *i*^th^ gene by $\mathbf{d}_{i}=(d_{i1},d_{i2},\cdots)$ and a count vector of the *i*^th^ gene by $\mathbf{f}_{i}=(f_{i1},f_{i2},\cdots)$. $d_{ij}$stands for the base pair residue of the *j*^th^ insertion site in the *i*^th^ gene. $f_{ij}$ is the insertion count at the *j*^th^ insertion site of the *i*^th^ gene. A site vector records where insertions are in a gene - the geometric location of each individual insertion. A count vector records the number of insertions per site per gene - the strength of an insertion at each insertion site. We denote the middle base pair residue of the *i*^th^ gene by $m_{i}$ and the gene length of the *i*^th^ gene by $L_{i}$. We introduce the following integration function for combining two-dimensional information into a genotype variable

| $g(\mathbf{f}_{i},\mathbf{d}_{i},L_{i})\mapsto x_{i}$ | (S2) |
| --- | --- |

where $L_{i}$ is used for normalisation, i.e. removing the effect of variable gene length on the formation of a genotypic variable. In addition to the two dimensions of $\mathbf{d}_{i}$ and $\mathbf{f}_{i}$, one more dimension is gene length. There have been different opinions regarding the treatment of the insertions, for example, TraDIS considers only insertions sites^5^ and ESSENTIALS considers only insertion counts^6^. Having understood that the insertion sites at the distal ends may not disrupt gene function as we have discussed in the main text of the paper, we consider combining the site dimension information with gene length information to generate a meaningful dimension in which the distal end effect can be treated appropriately. We introduce a novel ideal called a relative distance to the distal ends (RD) which is defined as below

| $r_{ij}=1-\frac{\vert m_{i}-d_{ij}\vert}{L_{i}/2}$ | (S3) |
| --- | --- |

where $|x|$ stands for absolute value of *x*. There is no doubt that $0\leq r_{ij}\leq1$. The importance of this novel idea is that it employs a quantitative measurement to reveal the significance of mutational position, i.e. positional significance on gene mutation. A small or a large RD certainly means different things. In addition, this variable removes the impact of variable gene length and importantly makes every individual RD comparable across genes. An individual RD can indicate its insertion position in relation to the distal ends no matter whether a gene is shorter or longer. If an insertion approaches the distal ends,$r_{ij}\to0$. If an insertion approaches the central area of a gene,$r_{ij}\to1$. Each gene then has a RD vector $\mathbf{r}_{i}=(r_{i1},r_{i2},\cdots)$ for multiple insertion sites per gene. The length of the vector of a gene varies depending how many insertions the gene has. The integration function is then simplified as

| $g(\mathbf{f}_{i},\mathbf{r}_{i})\mapsto x_{i}$ | (S4) |
| --- | --- |

Note that $|\mathbf{f}_{i}|\text{ }\geq\text{ }|\mathbf{r}_{i}|$, here two bars stand for the length of a vector. The equal sign exists only when the count is one for all insertion sites. The function integrates a vector of RD values to a single genotype variable ($x_{i}$) for each individual gene. Therefore, after this process, one gene should have one unique feature. This integration function therefore takes into account the interplay between different insertions in a gene for gene mutation prediction. We propose a simple integration function in this study

| $x_{i}^{R}=g(\mathbf{f}_{i},\mathbf{r}_{i})=\sum_{j=1}^{n_{i}} f_{ij}r_{ij}$ | (S5) |
| --- | --- |

where $n_{i}$ is the number of insertion sites of the *i*^th^ gene. In ESSENTIALS^2^, $r_{ij}\equiv1$ and Eq (S5) becomes , where $\hat{f}_{ij}$ is a corrected insertion count using a regression model. In most situations $\hat{f}_{ij}\approx f_{ij}>>1$ if noise count is removed as we discuss in Supplementary B. In TraDIS^1^, $r_{ij}\equiv1$ and $f_{ij}\equiv1$ and Eq (S5) becomes $x_{i}^{T}=\log_{2}n_{i}$ where the logarithm transformation is used.

RD increases the discrimination power between mutational and non-mutational genes. What we need to prove here is to show whether the difference between the feature of a non-mutational (essential) gene and the feature of a mutational (non-essential) gene can be enlarged by RD. We refer to this as the differential feature. Suppose we have two genes. Gene A is mutational and gene B is non-mutational. Suppose $n_{A}=n_{B}$ and $f_{A}=f_{B}$. In addition, the minimum RD for mutational gene is denoted by $\alpha$ and the maximum RD for non-mutational gene is denoted by$\beta$. Because gene A is mutational and gene B is non-mutational, $\sum_{j=1}^{n_{A}} r_{A,j}>>\sum_{j=1}^{n_{B}} r_{B,j}$ if we assume that insertions at distal ends hardly disrupt gene function. The differential feature of TraDIS is calculated as

| $\Delta x_{AB}^{T}=\left\vert\log_{2}n_{A}-\log_{2}n_{B}\right\vert=0$ | (S6) |
| --- | --- |

It can be seen that the differential feature calculated for two genes using the TraDIS approach is zero, meaning that TraDIS cannot discriminate between these two genes although they belong to different categories. For ESSENTIALS, the differential feature is calculated as

|  | (S7) |
| --- | --- |

Although using regression to correct insertion counts, $f_{ij}$ and $\hat{f}_{ij}$ should not be very different. It can be seen that ESSENTIALS is also unable to discriminate properly between these two genes. For RD, the differential feature value is calculated as

|  | (S8) |
| --- | --- |

It can be seen that if $\alpha-\beta>0$, the differential feature of RD is larger than the differential features of other two approaches.

The above discussion is based on Eq (S5), which employs a simple integration function between a site vector and a count vector. Due to noise in the data, this simple integration needs further revision. We consider a convolution model for the integration function in this work. The model estimates an exponential density based on the RD density

| $\kappa(r)=\beta(1-e^{-\beta r})$ | (S9) |
| --- | --- |

where $\beta$ is a parameter to be estimated and *r* is a vector of all RD values genome-wise. It can be seen that $\kappa(r)\to0$ when $r\to0$ and $\kappa(r)\to\beta$ when $r\to1$. The density function is estimated for all RD values leading to a function as $\kappa(r)$. For an individual gene with $n_{i}$ insertion sites, we have calls to the density function $\kappa(r)$, i.e. $\kappa(\mathbf{r}_{i})=\left(\kappa(r_{i1}),\kappa(r_{i2}),\cdots,\kappa(r_{in_{i}})\right)$. In our design, the integration between insertion location and insertion count is enhanced using these densities $\kappa(\mathbf{r}_{i})$,

| $x_{i}=\sum_{j=1}^{n_{i}} r_{ij}\kappa(r_{ij})$ | (S10) |
| --- | --- |

This density function will further penalise the distal end insertions.

**Distal Effect Model algorithm**

After the genotype variable (mutation feature) has been derived from the integration function using Eq (S10), we are required to consider how to model a mapping function between the genotype variable (*X*) and phenotype variable (*T*). We consider a mixture of Gammas for gene mutation classification. We formulate a Gamma mixture with two components following TraDIS^1^ and use the posterior probability for decision-making. The mixture of two Gammas is defined as

| $f(x_{i}\vert\theta)=w_{1}G(x_{i}\vert\alpha_{1},\beta_{1})+w_{2}G(x_{i}\vert\alpha_{2},\beta_{2})$ | (S11) |
| --- | --- |

where component one (parameterised by $w_{1},\text{ }\alpha_{1},\text{ }\beta_{1}$) with a low mean is for non-mutational (essential) genes and component two (parameterised by $w_{2},\text{ }\alpha_{2},\text{ }\beta_{2}$) is for mutational (non-essential) genes. A likelihood function is defined as

| $P(X\vert\theta)=\prod_{i=1}^{N} f(x_{i}\vert\theta)$ | (S12) |
| --- | --- |

A maximum likelihood training procedure is used to estimate model parameters$\theta=(w_{1},\text{ }\alpha_{1},\text{ }\beta_{1},w_{2},\text{ }\alpha_{2},\text{ }\beta_{2})$. TraDIS^1^ had two features that we think inappropriate. It used the likelihood value to identify non-mutational (essential) genes and it only used insertion sites for the prediction. We calculate posterior probability

| $P(\text{non}-\text{mutation}\vert x_{i})=\frac{w_{1}G(x_{i}\vert\alpha_{1},\beta_{1})}{w_{1}G(x_{i}\vert\alpha_{1},\beta_{1})+w_{2}G(x_{i}\vert\alpha_{2},\beta_{2})}$ | (S13) |
| --- | --- |

where $P(\text{non}-\text{mutation}|x_{i})$ reads as the posterior probability that the $x_{i}$ (genotype variable value for the *i*^th^ gene) belongs to the class of non-mutational genes.

**False discovery rate control**

We convert the posterior probability to a false discovery rate based on previous work^7^. Genes will be classified as non-mutational ones if their false discovery rates are less than a critical value such as 0.01.

**Tight cluster for noise remove**

It is believed that high-throughput sequencing data may contain noise due to counting error and sample variation^8^. Normally low insertion count per site is treated as noise. The existence of the low insertion count per site will make mutational gene prediction difficult supposing such noise appears close to the middle base pair residue of a gene. Their RDs are certainly approaching one, $r_{ij}\to1$ if $\left|m_{i}-d_{ij}\right|\to0$. Their existence may make the mutation feature for some genes unnecessarily high. Thus the existence of these noise insertions will make the mapping function estimate difficult. The solution is to remove noise counts. Therefore all insertion counts were pooled gene-wise. They were transformed using the logarithm, but genes without insertion were excluded from the analysis. In this paper, we used three methods to determine the boundary between noise insertion and non-noise insertion counts. We used the tight cluster approach^9^.

**Reference**

1. Parkhill, J., *et al*. Genome sequence of Yersinia pestis, the causative agent of plague. *Nature*. **413**, 523-527 (2001).

2. Ford, D. C., *et al*. Construction of an inducible system for the analysis of essential genes in Yersinia pestis. *J Microbiological Methods*. **100**, 1-7 (2014).

3. Taylor, V. L., *et al*. Oral immunization with a dam mutant of Yersinia pseudotuberculosis protects against plague. *Microbiology* **151**, 1919-1926 (2005).

4. Maxson, M. E. and Darwin, A. J. Identification of inducers of the Yersinia enterocolitica phage shock protein system and comparison to the regulation of the RpoE and Cpx extracytoplasmic stress responses. *J Bacteriology*. **186**, 4199-4208 (2004).

5. Langridge, G., et al. Simultaneous assay of every Salmonella Typhi gene using one million transposon mutants. *Genome Res*. **19**, 2308-16 (2009).

6. Zomer, A., et al. ESSENTIALS: software for rapid analysis of high throughput transposon insertion sequencing data. *PLoS One*. **7**, e43012 (2012).

7. Storey, J. D. A direct approach to false discovery rates. *J. R. Stat. Soc.* **64**, 479-98 (2002).

8**.** Sims, R. J., *et al*. Sequencing depth and coverage: key considerations in genomic analyses. *Nature Review Genetics*. **15**, 121-32 (2014).

9. Yang, Z., Yang, Z. R. Prediction of heterogeneous differential genes by detecting outliers to a Gaussian tight cluster. *BMC Bioinformatics*. **14**, 81 (2013).
